# The advanced treatment of hydrogen bonding in quantum crystallography

**DOI:** 10.1107/S1600576721001126

**Published:** 2021-04-16

**Authors:** Lorraine A. Malaspina, Alessandro Genoni, Dylan Jayatilaka, Michael J. Turner, Kunihisa Sugimoto, Eiji Nishibori, Simon Grabowsky

**Affiliations:** a Universität Bern, Departement für Chemie, Biochemie und Pharmazie, Freiestrasse 3, 3012 Bern, Switzerland; b Universität Bremen, Fachbereich 2 – Biologie/Chemie, Institut für Anorganische Chemie und Kristallographie, Leobener Strasse 3, 28359 Bremen, Germany; c Université de Lorraine and CNRS, Laboratoire de Physique et Chimie Théoriques (LPCT), UMR CNRS 7019, 1 Boulevard Arago, 57078 Metz, France; d The University of Western Australia, School of Molecular Sciences, 35 Stirling Highway, Perth, WA 6009, Australia; e Japan Synchrotron Radiation Research Institute/Diffraction and Scattering Division, 1-1-1 Kouto, Sayo-cho, Sayo-gun, Hyogo 679-5198, Japan; f Institute for Integrated Cell-Material Sciences (iCeMS), Kyoto University, Yoshida-Ushinomiya-cho, Sakyo-ku, Kyoto 606-8501, Japan; g Department of Physics, Faculty of Pure and Applied Sciences, Tsukuba Research Center for Energy Materials Science (TREMS), University of Tsukuba, Tsukuba, Japan

**Keywords:** quantum crystallography, Hirshfeld atom refinement, X-ray constrained wavefunction fitting, electron density, hydrogen bonding

## Abstract

Although hydrogen bonding is one of the most important motifs in chemistry and biology, H-atom parameters are especially problematic to refine against X-ray diffraction data. New developments in quantum crystallography offer a remedy. This article reports how hydrogen bonds are treated in three different quantum-crystallographic methods: Hirshfeld atom refinement (HAR), HAR coupled to extremely localized molecular orbitals and X-ray wavefunction refinement. Three different compound classes that form strong intra- or intermolecular hydrogen bonds are used as test cases: hydrogen maleates, the tripeptide l-alanyl-glycyl-l-alanine co-crystallized with water, and xylitol. The differences in the quantum-mechanical electron densities underlying all the used methods are analysed, as well as how these differences impact on the refinement results.

## Abstract 2

Hydrogen-bonding parameters are modelled with different methods of quantum crystallography. The underlying model assumptions are analysed and related to the refinement results.

## 1. Introduction   

Hydrogen bonding is the most important intermolecular interaction and as such an essential structure- and reactivity-determining motif in chemistry, biology, catalysis, materials science and many other fields (Arunan *et al.*, 2011 ▸; Fonseca Guerra *et al.*, 1999 ▸; Pimentel & McClellan, 1971 ▸; Hibbert & Emsley, 1990 ▸; Grabowski, 2006 ▸; Desiraju & Steiner, 2001 ▸). In crystallography, hydrogen bonding is the key force in stabilizing molecular assemblies (Etter *et al.*, 1990 ▸; Steiner, 2002 ▸). In fact, hydrogen bonds in carefully chosen small-molecule crystal structures can be regarded as model interactions present in proton-transfer reactions and molecular recognition processes of larger biological systems (Overgaard *et al.*, 1999 ▸; Schiøtt *et al.*, 1998 ▸; Shi *et al.*, 2015 ▸; Grabowsky *et al.*, 2013 ▸). Therefore, it is important to be able to model H-atom positions, atomic displacement parameters and derived properties accurately and precisely from crystallographic diffraction experiments.

Neutron-diffraction experiments are the gold standard for the accurate and precise localization of H atoms in crystal structures. In standard X-ray diffraction experiments, H atoms are more difficult to locate and, if refined freely, bond distances involving H atoms are usually too short by about 0.1 Å (Cooper *et al.*, 2010 ▸). The reason for this shortening in standard X-ray refinements is that the single electron of the H atom is a valence electron which is shifted into the chemical bond. The problem can be solved by replacing the model of spherical atoms (independent atom model) with a more suitable electron-density model that incorporates the effect of chemical bonding, *i.e.* the nonsphericity of the atomic electron-density distribution in bonded atoms. We note that hydrogen-bonded systems are prone to vibrate anharmonically. However, in this work we only investigate the model improvements caused by nonspherical H-atom treatment, still in the harmonic approximation.

Several such nonspherical atom models exist among the techniques of quantum crystallography (Grabowsky *et al.*, 2017 ▸, 2020 ▸; Genoni *et al.*, 2018 ▸; Genoni & Macchi, 2020 ▸). In multipole modelling (MM) (Hansen & Coppens, 1978 ▸), H-atom positions and anisotropic displacement parameters (ADPs) are normally not refined, unless very high quality data are used (Zhurov *et al.*, 2011 ▸). Instead, bonds involving H atoms and hydrogen ADPs are often set to values derived from neutron-diffraction experiments (Allen & Bruno, 2010 ▸; Madsen, 2006 ▸) and kept fixed during the refinement (Hoser *et al.*, 2009 ▸; Köhler *et al.*, 2019 ▸). Alternatively, multipole parameters can be transferred from databanks (either constructed from theoretical calculations or averaged over experimental multipole refinements) and fixed during the refinement of positions and ADPs, which leads to more stable refinements of H-atom parameters (Dittrich *et al.*, 2005 ▸; Dadda *et al.*, 2012 ▸; Bąk *et al.*, 2011 ▸; Jha *et al.*, 2020 ▸).

In Hirshfeld atom refinement (HAR) (Jayatilaka & Dittrich, 2008 ▸; Capelli *et al.*, 2014 ▸), H-atom positions and sometimes also ADPs can be refined freely, and the results agree favourably with those from neutron diffraction (Woińska *et al.*, 2016 ▸; Fugel *et al.*, 2018 ▸; Sanjuan-Szklarz *et al.*, 2020 ▸). HAR has also been tested for strong hydrogen bonds (Woińska *et al.*, 2014 ▸). A drawback of the HAR method is its reduced speed in comparison with MM methods, since it relies on the repeated calculation of molecular wavefunctions. To overcome this drawback, it was coupled to libraries of extremely localized molecular orbitals (ELMOs) (Meyer, Guillot, Ruiz-Lopez & Genoni, 2016 ▸; Meyer, Guillot, Ruiz-Lopez, Jelsch & Genoni, 2016 ▸; Meyer & Genoni, 2018 ▸), giving rise to the HAR-ELMO method (Malaspina *et al.*, 2019 ▸). H-atom treatment and comparison of H-atom parameters for HAR-ELMO have been described by Malaspina *et al.* (2019 ▸).

In HAR, HAR-ELMO and multipole database techniques, also called the transferable aspherical atom model, the electron density is calculated theoretically or transferred from a databank and then fixed during the refinement of atomic positions and displacement parameters. In contrast, in multipole modelling, the electron density is refined together with positions and displacement parameters. Therefore, the way in which H atoms are treated impacts directly on the distribution and topology of the refined multipolar electron density (Hoser *et al.*, 2009 ▸; Roversi & Destro, 2004 ▸; Madsen *et al.*, 2004 ▸). As an alternative way of extracting the electron-density distribution from the X-ray diffraction experiment, X-ray wavefunction refinement (XWR) (Woińska *et al.*, 2017 ▸) combines HAR with X-ray constrained wavefunction (XCW) fitting (Jayatilaka, 1998 ▸; Jayatilaka & Grimwood, 2001 ▸; Grimwood & Jayatilaka, 2001 ▸). The impact of H-atom treatment on the fitted electron density in XWR is less well studied. Malaspina *et al.* (2020 ▸) started investigating in detail the influence of H-atom dis­place­ment parameters on both geometry from HAR and electron density from XWR for strong intramolecular hydrogen bonds. In the present study, we continue the previous work, in particular by analyzing how different ways of treating the H atoms (namely, using HAR, HAR-ELMO and XWR) impact on positions, ADPs and electron density parameters of H atoms involved in inter- and intramolecular hydrogen bonds.

For HAR, the recently introduced software *lamaGOET* (Malaspina *et al.*, 2021 ▸) allows users to interface the quantum-crystallographic program *Tonto* (Jayatilaka & Grimwood, 2003 ▸) with quantum-chemical software such as *Gaussian* (Frisch *et al.*, 2016 ▸). In this study, such ‘*Gaussian*-HARs’ were performed on compounds containing the hydrogen maleate anion, where an H atom bridges two O atoms in a strong and short intramolecular hydrogen bond. Depending on the counter-cation, the position of the H atom can shift from being perfectly symmetric to being asymmetric (Malaspina *et al.*, 2017 ▸). Such a bridging H-atom position is an especially difficult situation to model using X-ray diffraction data.

In HAR-ELMO (Malaspina *et al.*, 2019 ▸), the basic assumption of the model, namely the strict localization of the ELMOs, may impact on such regions where electronic delocalization plays a role, *e.g.* in the amide or carboxylate regions of peptides. The underlying electron densities in the HAR-ELMO treatment have been analyzed (Meyer, Guillot, Ruiz-Lopez & Genoni, 2016 ▸; Meyer, Guillot, Ruiz-Lopez, Jelsch & Genoni, 2016 ▸) and quantum mechanics/extremely localized molecular orbital (QM/ELMO) embedding techniques have been developed to improve the electron-density analysis for such regions (Macetti & Genoni, 2019 ▸, 2020 ▸; Macetti *et al.*, 2020 ▸). However, for HAR-ELMO the impact of the ELMO approximation on refined geometric and displacement parameters has not been studied yet. Especially in regions of intermolecular interactions such as hydrogen bonds, the shape of the electron-density distribution is important for the refinement results. Therefore, here we demonstrate a HAR-ELMO treatment of the tripeptide l-alanyl-glycyl-l-alanine (AGA), co-crystallized with one hydrogen-bonded water molecule, and compare deformation electron densities.

In XWR, the effect of the experimental constraint on the wavefunction becomes very important when strong intermolecular interactions significantly polarize the electron density of the molecule in the crystal field compared with the isolated case (Ernst *et al.*, 2020 ▸). Therefore, we present a full XWR treatment of xylitol, a molecule that is involved in many strong intermolecular hydrogen bonds in its crystal packing, and investigate the effect of the polarization on the electron-density distribution as captured by the XCW fitting procedure.

## 2. Experimental details   

The four different data sets used in this study were taken from previously published and deposited high-resolution low-temperature single-crystal X-ray diffraction experiments. For the two compounds 8-hydroxyquinolinium hydrogen maleate (8HQ HMal) and magnesium bis(hydrogen maleate) hexahydrate (Mg HMal) we used the same synchrotron data, measured at beamline BL02B1 of SPring-8, that were used before by Malaspina *et al.* (2020 ▸). The crystallographic structure factors of AGA were taken from Förster *et al.* (2007 ▸). They were measured at the Swiss Light Source synchrotron, beamline X10SA. The data of xylitol are laboratory Mo *K*α data, reported by Madsen *et al.* (2004 ▸). Pertinent crystallographic and measurement details are reiterated in Tables 1 ▸ and 2 ▸.

The hydrogen maleate compounds were subjected to a HAR using *Tonto* only (‘normal HAR’), and to a HAR using *lamaGOET* as an interface to *Gaussian* for the quantum-chemical calculations and to *Tonto* for the partitioning and least-squares refinement. From now on we will refer to the latter kind of HAR as *Gaussian*-HAR. In all refinements, the crystal field was simulated with cluster charges within a radius of 8 Å around the central asymmetric unit. More refinement details (such as the different levels of theory used) and the refinement results are discussed in Section 3.1. CIFs are deposited with the Cambridge Structural Database (CSD) and can be obtained via CCDC deposition numbers 1987762 (8HQ HMal) and 1987825 (Mg HMal). They are also included as supporting information for this article. More details on the software *lamaGOET* and the realization of *Gaussian*-HARs are given by Malaspina *et al.* (2021 ▸).

The AGA refinements serve as an example for a HAR-ELMO application on a peptide. The recently introduced HAR-ELMO procedure (Malaspina *et al.*, 2019 ▸) uses the software *lamaGOET* to pass the ELMO-derived wavefunction from the *ELMOdb* software to the refinement software *Tonto. lamaGOET* is compatible with the ELMO nomenclature, also for tailor-made residues. Details of the software behind HAR-ELMO are discussed by Malaspina *et al.* (2019 ▸, 2021 ▸). The HAR-ELMO treatment performed for AGA used transferred ELMOs expanded on the 6-311G(*d*,*p*) basis set, while the traditional HAR was based on repeated HF/6-311G(*d*,*p*) wavefunction calculations. No cluster charges were used in either HAR or HAR-ELMO. Refinement results are discussed in Section 3.2. CIFs are deposited in the CSD and can be obtained via 1987828 or from the supporting information.

For xylitol, XWR was performed as a sequence of HAR and XCW fitting in *Tonto*, mediated, facilitated and controlled by the software *lamaGOET* (Malaspina *et al.*, 2021 ▸). Both HAR and XCW fitting were carried out using the HF/6-311G(*d*,*p*) level of theory. For HAR, an 8 Å surrounding cluster of point charges and dipoles was used to simulate crystal-field effects and to obtain accurate positions of H atoms involved in hydrogen bonding (Fugel *et al.*, 2018 ▸). For the XCW fitting part, this cluster was not used to probe whether the XCW fitting procedure incorporates the crystal field effect into the isolated-molecule wavefunction ansatz. The corresponding CIFs are deposited with the CSD under deposition number 1987830 and in the supporting information. In addition, a theoretical single-point calculation using the HAR geometry was performed at the B3LYP/6-311G(*d*,*p*) level of theory in *Tonto*.

## 3. Results and discussion   

### 3.1. Hirshfeld atom refinement of hydrogen maleates   

**Figure 1:**
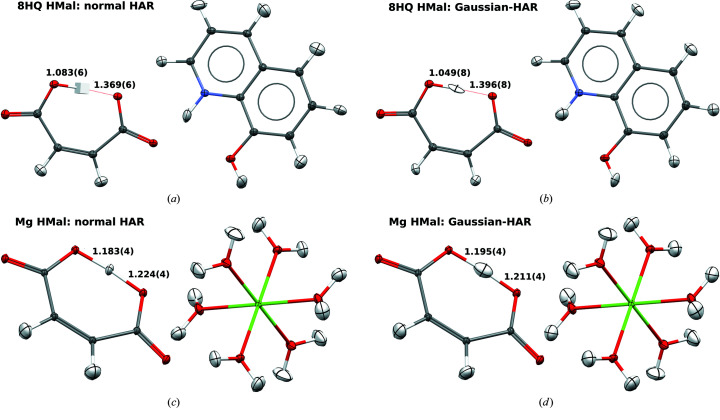
First row: refined structure of 8HQ HMal, including O—H bond distances in Å. (*a*) HAR performed exclusively with *Tonto* (HF/def2-TZVP). (*b*) HAR performed with *Gaussian* and *Tonto* interfaced through *lamaGOET* [B3PW91/6-311++G(*d*,*p*)]. The neutron-diffraction-derived distances are 1.072 (3) and 1.378 (4) Å (Malaspina *et al.*, 2017 ▸). Second row: refined structure of Mg HMal, including O—H bond distances in Å. (*c*) HAR performed exclusively with *Tonto* (HF/def2-TZVP). (*d*) HAR performed with *Gaussian* and *Tonto* interfaced through *lamaGOET* [B3PW91/6-311++G(*d*,*p*)]. The neutron-diffraction-derived distances are 1.1873 (16) and 1.2181 (16) Å (Malaspina *et al.*, 2017 ▸). All ADPs are at 50% probability level. The cube at the hydrogen position in (*a*) denotes a non-positive-definite hydrogen ADP matrix. Graphics produced with the software *Mercury* (Macrae *et al.*, 2020 ▸).

The compound class of hydrogen maleates is especially suited to challenging and probing HAR because the hydrogen maleate anion presents a H atom in a bridging position that closes the hydrogen maleate anion into a seven-membered ring structure compatible with a strong intramolecular-resonance-assisted hydrogen bond (Fig. 1 ▸) (Gilli & Gilli, 2000 ▸; Mahmudov & Pombeiro, 2016 ▸). Woińska *et al.* (2014 ▸) have shown previously that, for the example of l-phenylalaninium hydrogen maleate, HAR is able to accurately reproduce the symmetric H-atom position, referenced to results from neutron diffraction. In further neutron-diffraction studies, we have demonstrated how the identity of the counter-cation influences the H-atom position in the intramolecular hydrogen bond via the crystal field, being symmetric, asymmetric or intermediate (Malaspina *et al.*, 2017 ▸). Such small yet significant differences in the H-atom position in the same anion are only influenced by intermolecular interactions and are extremely hard to model on the basis of X-ray data. However, Malaspina *et al.* (2020 ▸) managed to do so with HAR for an extended series of hydrogen maleates, including the two compounds studied here: 8HQ HMal and Mg HMal.

It was shown in the study on HAR and l-phenylalaninium hydrogen maleate (Woińska *et al.*, 2014 ▸) that the free anisotropic refinement of the H atom in the intramolecular hydrogen bond led to an accurate reproduction of its symmetric position. It was also shown that even if the hydrogen ADP matrix becomes non-positive definite (NPD), the accuracy of the *X*—H bond distance is not diminished (Woińska *et al.*, 2016 ▸). However, since NPD hydrogen ADP matrices are physically meaningless (Dittrich *et al.*, 2017 ▸), we investigated other ways of estimating the hydrogen ADPs or we refined the atom isotropically (Malaspina *et al.*, 2020 ▸). Here, we want to test the influence of changing the method and the basis set on the H-atom position and the refined ADPs – a question left open by Malaspina *et al.* (2020 ▸) because a quantum-crystallographic interface such as the new *lamaGOET* software was needed for such a study (Malaspina *et al.*, 2021 ▸).

For the 8HQ HMal structure [Fig. 1 ▸(*a*)], a normal HAR with the program *Tonto* using a recommended level of theory (HF/def2-TZVP; see Fugel *et al.*, 2018 ▸) produces an NPD hydrogen ADP matrix. Nevertheless, the O—H bond distances are accurate and agree with the neutron-diffraction-derived bond distances within two standard uncertainties (see caption of Fig. 1 ▸). To improve the ADP description in the 8HQ HMal structure, we have employed *lamaGOET* to modify the level of theory used in HAR. By exploiting *lamaGOET*’s interface to *Gaussian* (Malaspina *et al.*, 2021 ▸), HAR can now be performed with many density functional theory (DFT) exchange-correlation functionals, post-Hartree–Fock (post-HF) methods (Wieduwilt *et al.*, 2020 ▸) and basis sets that are not implemented in *Tonto*. This significantly improves the flexibility of HARs. Therefore, here we have chosen the level of theory B3PW91/6-311++G(*d*,*p*), where neither the method nor the basis set is available for a normal HAR in *Tonto*. We anticipated that the use of diffuse functions could improve the description of the H atom in the anionic hydrogen maleate. Indeed, the hydrogen ADP improved, as we no longer obtain an NPD hydrogen ADP matrix [Fig. 1 ▸(*b*)]. However, it is still skewed, showing that HAR is in general not a method for the accurate determination of hydrogen ADPs but mainly one for determining *X*—H distances (*cf*. Köhler *et al.*, 2019 ▸). The *Gaussian*-HAR-derived O—H bond distances agree with the neutron-diffraction results within just above three standard uncertainties.

Another problem that sometimes occurs in *Tonto*-based HARs is related to linear dependencies when spherical ions are treated. Owing to its coordination to six water molecules, we were able to obtain a converged normal HAR in *Tonto* for the magnesium cation in Mg HMal at the HF/def2-TZVP level of theory [Fig. 1 ▸(*c*)]. All hydrogen ADPs look reasonable, and the O—H distances agree with those derived from neutron diffraction within a single standard uncertainty (see caption of Fig. 1 ▸). Variation of the level of theory [B3PW91/6-311++G(*d*,*p*)] influences the refinement results [Fig. 1 ▸(*d*)]. The ellipsoid associated with the hydrogen ADPs in the intramolecular hydrogen bond has become larger and more stretched along the bond vector, which also slightly changes the O—H bond distances. However, they are still accurate, being well within two standard uncertainties of the neutron-diffraction-derived results.

In the cases discussed above, the level of theory has a nonnegligible influence on ADPs and bond lengths only for the H atom involved in the strong hydrogen bond, while the parameters of the other covalently bonded H atoms in C—H, N—H and O—H bonds are unaffected. Since in the cases reported in Fig. 1 ▸ we have varied two parameters at once (namely, method and basis set), we now extend the series of HAR models by a normal HAR at the B3LYP/def2-TZVP level of theory and a *Gaussian*-HAR at the B3PW91/def2-TZVP level of theory. The results are summarized in Table 3 ▸ and depicted in terms of refined molecular structures and residual electron density plots in the supporting information.

The only NPD hydrogen ADP matrix occurs for the HF refinement of 8HQ HMal. Regardless of the DFT functional, the ADP matrix of atom H1 in the strong intramolecular hydrogen bond of the hydrogen maleate anion is always positive definite and the corresponding O1—H1 and O2—H1 distances are closer to the reference values from neutron diffraction (Table 3 ▸). This qualitative difference between HF and DFT refinements is also reflected in the χ^2^ value listed in Table 3 ▸ for both compounds 8HQ and Mg HMal, but not in the *R* values and min/max residual density values which are very similar among the refinements. A similar observation, but less pronounced, can be made when comparing the def2-TZVP basis set with the 6-311++G(*d*,*p*) basis set. The χ^2^ value becomes higher again, whereas the other quality indicators are not indicative of a significant difference. This means that diffuse functions do not positively influence the H-atom treatment in the anionic hydrogen maleate. In summary, both B3LYP/def2-TZVP and B3PW91/def2-TZVP HARs perform better than the HF/def2-TZVP and B3PW91/6-311++G(*d*,*p*) HARs.

**Figure 2:**
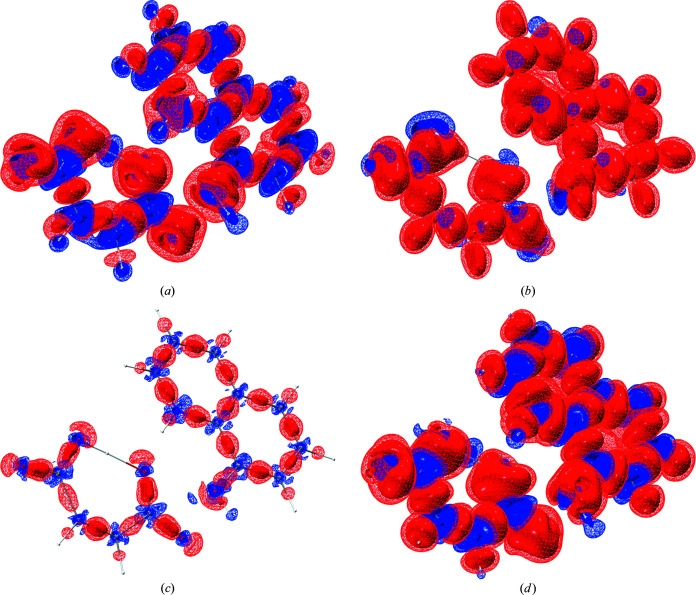
Differences of the theoretical deformation densities underlying 8HQ HMal refinements at different levels of theory (blue = positive, red = negative; isosurfaces, wireframe at 0.025 e Å^−3^ and solid at 0.05 e Å^−3^). (*a*) B3LYP/def2-TZVP minus HF/def2-TZVP, depicting the effect of electron correlation; (*b*) B3PW91/def2-TZVP minus B3LYP/def2-TZVP, depicting the effect of different hybrid DFT functionals; (*c*) B3PW91/6-311++G(*d*,*p*) minus B3PW91/def2-TZVP, depicting the basis-set dependency; (*d*) B3PW91/6-311++G(*d*,*p*) minus HF/def2-TZVP, depicting the superposition of all effects. The grid files containing the individual deformation density distributions are based on the final geometries after refinement and thus they slightly deviate from each other. Therefore, the difference deformation densities shown here are not exactly identical to the corresponding difference electron densities, but they are qualitatively very similar. The molecular structures shown are always those of the first method mentioned in the differences. Graphics were produced with the program *VMD* (Humphrey *et al.*, 1996 ▸).

*lamaGOET* offers the possibility to test all the levels of theory available in *Gaussian* to find the most suitable level of theory for a particular compound, which was not possible before in *Tonto*. Therefore, in a final step of this section of the study we compare the theoretical electron densities underlying the different HARs presented in Table 3 ▸. Figs. 2 ▸ and 3 ▸ show such comparisons for compounds 8HQ and Mg HMal, respectively.

The findings are identical for both compounds, so the discussion of Figs. 2 ▸ and 3 ▸ can be unified. Figs. 2 ▸(*a*) and 3 ▸(*a*) show the effect of changing the method from HF to DFT. The effect is large and systematic. Electron density is shifted from the bonds into the core regions of all the atoms including the H atoms. This is the known impact of electron correlation on the electron density distribution of molecules (Wiberg *et al.*, 1992 ▸), which will be discussed in more detail with respect to XCW fitting in Section 3.3. Here, this shift of electron density is responsible for the improvements in the refinements found according to Table 3 ▸.

The difference between the DFT methods B3LYP and B3PW91 depicted in Figs. 2 ▸(*b*) and 3 ▸(*b*) is also large. In contrast to the difference between HF and DFT, electron density is not accumulated in the core regions, only redistributed in the valence region. The effect on the refined atom positions, ADPs and refinement statistics is clearly less significant than in the case of HF versus DFT.

Figs. 2 ▸(*c*) and 3 ▸(*c*) show the redistribution of electron density due to the change of the basis set. The effect is much smaller than the effect of changing the method discussed in the previous paragraphs. However, qualitatively the shift of electron density is from the bonding and lone-pair valence regions into the core regions, similar to the HF versus DFT difference. This influences the refinement as seen in Table 3 ▸, but to a smaller extent than the HF versus DFT difference. Figs. 2 ▸(*d*) and 3 ▸(*d*) show the combined effect of changing the method from HF to DFT and from an Ahlrichs to a Pople triple-zeta basis set, reflected in the differences between the molecular structures depicted in Fig. 1 ▸.

### 3.2. HAR-ELMO treatment of the tripeptide AGA   

**Figure 4:**
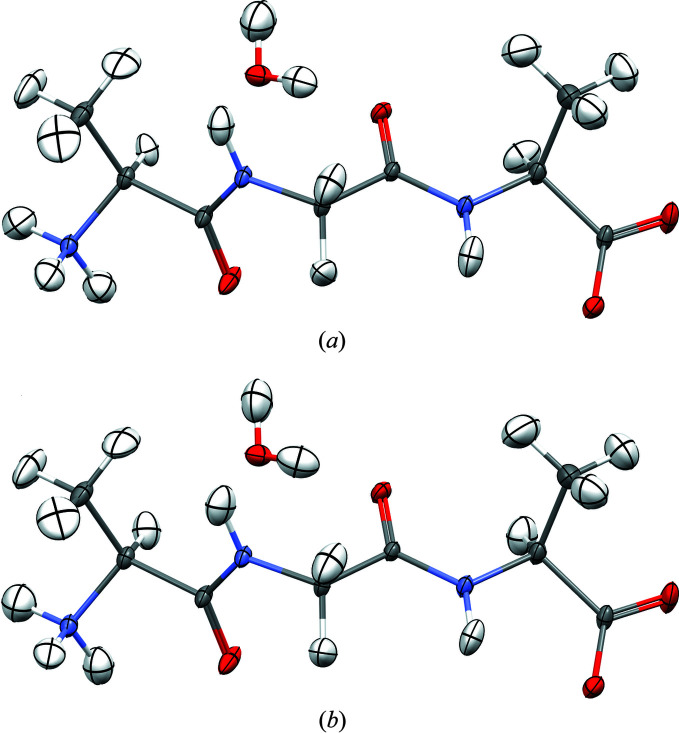
Molecular structures of AGA refined using (*a*) HAR and (*b*) HAR-ELMO. All ADPs are at 50% probability level. Graphics produced with the software *Mercury* (Macrae *et al.*, 2020 ▸).

In agreement with the test cases in the original publication (Malaspina *et al.*, 2019 ▸), the geometries and ADPs, including hydrogen ADPs, are virtually identical for the HAR and HAR-ELMO treatments at the same levels of theory (Fig. 4 ▸). Also in terms of statistics (Table 4 ▸), the two refinements are very similar. However, HAR-ELMO always shows slightly worse values, which is expected since it includes an approximation not made in a normal HAR. Nevertheless, HAR-ELMO is faster by a factor larger than 4 (see last row in Table 4 ▸).

**Figure 5:**
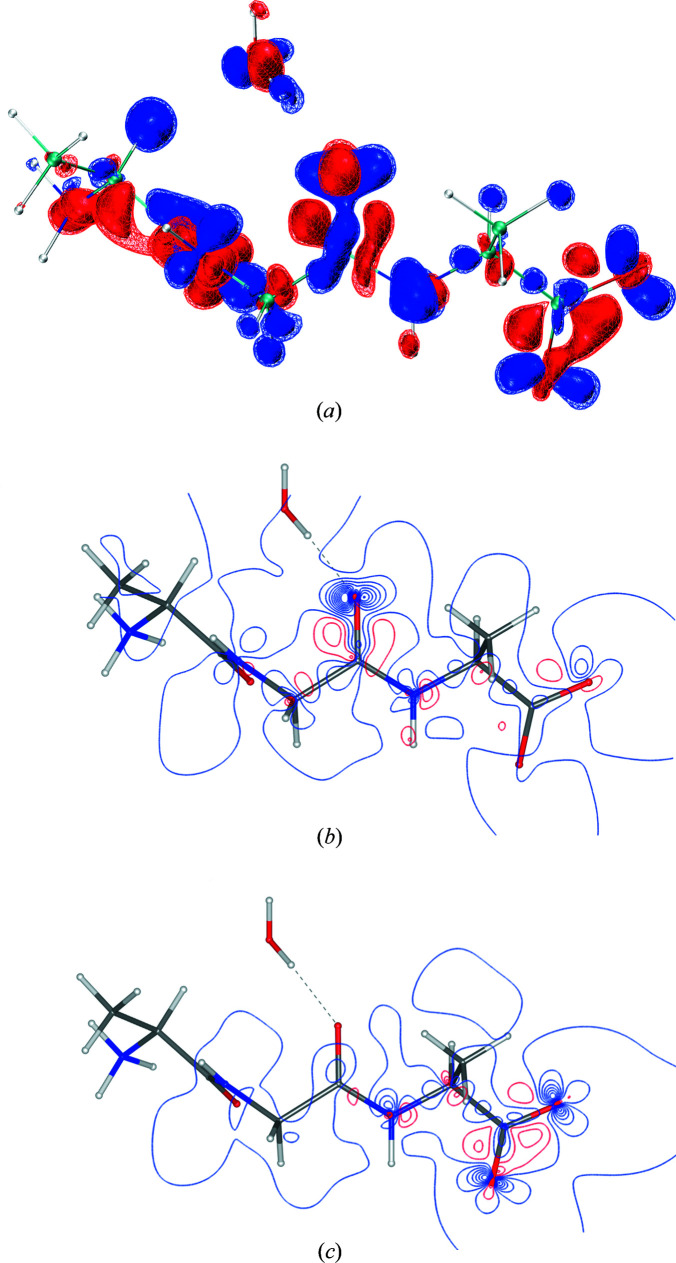
Difference deformation density plots of AGA: HAR-ELMO minus HAR (blue = positive, red = negative). (*a*) Isosurfaces, wireframe at 0.04 e Å^−3^ and solid at 0.05 e Å^−3^; (*b*) plane of an amide group, isocontour level: 0.05 e Å^−3^; (*c*) plane of the carboxylate group, isocontour level: 0.05 e Å^−3^. The grid files containing the individual deformation density distributions are based on the final geometries after refinement and thus they slightly deviate from each other. Therefore, the difference deformation densities shown here are not exactly identical to the corresponding difference electron densities, but they are qualitatively very similar. The molecular structures shown are those of the HAR-ELMO treatment. Graphics produced with the programs *VMD* for the 3D plot (Humphrey *et al.*, 1996 ▸) and *VESTA* for the 2D plots (Momma & Izumi, 2011 ▸).

Although the geometric and statistical results of HAR-ELMO treatment are very promising, especially for the treatment of H atoms in protein crystallography (Malaspina *et al.*, 2019 ▸), so far the influence of the model assumption (the extreme localization of the frozen molecular orbitals) on the electron-density distribution used in the refinement has not been addressed. Only some preliminary observations have been made in the supporting information of Malaspina *et al.* (2019 ▸) and in the article by Grabowsky *et al.* (2021 ▸). Here, we show the difference between the HAR and HAR-ELMO deformation electron densities of AGA as three- and two-dimensional maps (Fig. 5 ▸).

At the given isolevel, there are significant differences in the deformation densities between the two refinements. The blue colour means that there is more electron density concentrated in these regions for the HAR-ELMO case. The differences are most pronounced in the amide and the carboxylate regions, where resonance effects play a role. In particular, the description of the lone pairs is different, which is very pronounced for the oxygen atoms in the two-dimensional maps, but also occurs for the nitrogen-atom lone pairs that are perpendicular to the chosen cut planes. Since the lone pairs have more electron density in the HAR-ELMO model, the delocalization and hence charge redistribution are less pronounced, and presumably less realistic, in the HAR-ELMO model compared with the HAR model because of the strict localization and the lack of charge relaxation after the transfer.

Close to the oxygen cores, the difference electron density can become as large as 0.3 e Å^−3^. This has an effect on derived properties such as atomic charges and electrostatic potentials as already discussed (Meyer, Guillot, Ruiz-Lopez & Genoni, 2016 ▸; Meyer, Guillot, Ruiz-Lopez, Jelsch & Genoni, 2016 ▸; Meyer & Genoni, 2018 ▸). In particular, it was shown that the use of transferred ELMOs results in quite systematic overestimations of charges associated with the subunits of the system. This might have an important effect on modelling the geometries of systems in regions important for intermolecular interactions, which are crucial in molecular recognition processes of biological interest.

Owing to the co-crystallized water molecule, there is a strong intermolecular interaction in the asymmetric unit of AGA, namely the hydrogen bond from water to the carbonyl oxygen atom in the glycil unit (see Fig. 4 ▸). Fig. 5 ▸(*a*) shows that the two O atoms involved and also the water H atom in the hydrogen bond are modelled differently in the two approaches: the lone pairs and the H atom possess a larger electron density in the HAR-ELMO model, whereas the oxygen cores have less electron density in the HAR-ELMO model. In the HAR [Fig. 4 ▸(*a*)], the characteristics of the hydrogen bond are *d*(*D*—H) = 0.946 (10) Å, *d*(H⋯*A*) = 1.873 (10) Å, *d*(*D*⋯*A*) = 2.810 (2) Å, *a*(*D*—H⋯*A*) = 170.6 (9)°. For HAR-ELMO [Fig. 4 ▸(*b*)], they are *d*(*D*—H) = 0.907 (10) Å,* d*(H⋯*A*) = 1.910 (10) Å, *d*(*D*⋯*A*) = 2.809 (2) Å, *a*(*D*—H⋯*A*) = 170.9 (9)°. Hence, the bond distances involving the H atom are significantly different between the two refinements (0.04 Å, which corresponds to four standard uncertainties), whereas the bond angle is less affected. Such differences need to be taken into consideration when HAR-ELMO is used for protein crystallography in the future. To overcome this drawback, the QM/ELMO approach has recently been introduced (Macetti & Genoni, 2019 ▸; Macetti *et al.*, 2020 ▸). This new technique enables one to treat the most important part of a biological system (*e.g.* the active site of a protein or a region involved in important intermolecular interactions for mol­ecular recognition) at a higher level of theory, with the rest still described through transferred and frozen ELMOs. This will most likely solve the problem discussed in this paragraph and will undergo further studies within a future HAR-QM/ELMO approach.

### 3.3. X-ray wavefunction refinement of xylitol   

**Figure 6:**
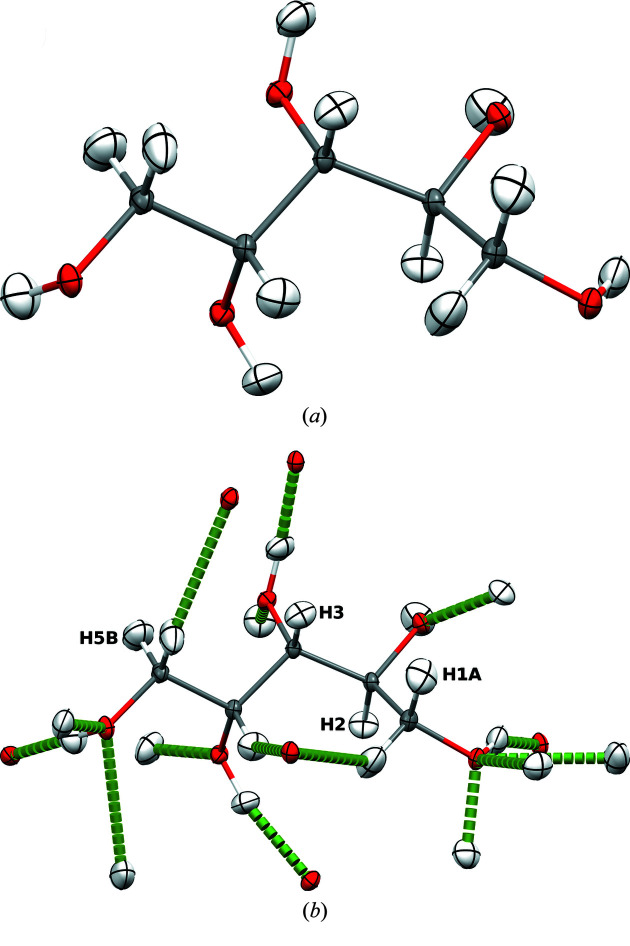
(*a*) HAR-derived molecular structure of xylitol. (*b*) Hydrogen-bonding network of xylitol. Only those H atoms that are not involved in hydrogen bonds are labelled. Criteria for identification of hydrogen bonds: maximum H⋯*A* distance range = sum of H and *A* van der Waals radii; *D*—H⋯*A* angle > 120°; donor and acceptor separated by more than three bonds. All ADPs are at 50% probability level. Graphics produced with the software *Mercury* (Macrae *et al.*, 2020 ▸).

The crystal structure of xylitol contains five hydroxy groups [Fig. 6 ▸(*a*)], and hence forms numerous O—H⋯O and C—H⋯O hydrogen bonds [see Fig. 6 ▸(*b*)]. All the O atoms are involved in hydrogen bonding as both donors and acceptors, and all but four of the 12 H atoms are hydrogen bonded; the ones not involved are labelled in Fig. 6 ▸(*b*). This means that crystal-field effects play an important role in the xylitol crystal structure. Previous studies have shown that crystal-field effects (polarization) together with electron correlation effects are the most important features that can be added to the single-molecule wavefunction through the variational procedure of the wavefunction fitting approach (Bytheway *et al.*, 2007 ▸; Bučinský *et al.*, 2016 ▸; Genoni *et al.*, 2017 ▸; Grabowsky *et al.*, 2021 ▸; Ernst *et al.*, 2020 ▸). This is further investigated here since we expect that the effect of fitting is pronounced in the strong crystal field of xylitol.

**Figure 7:**
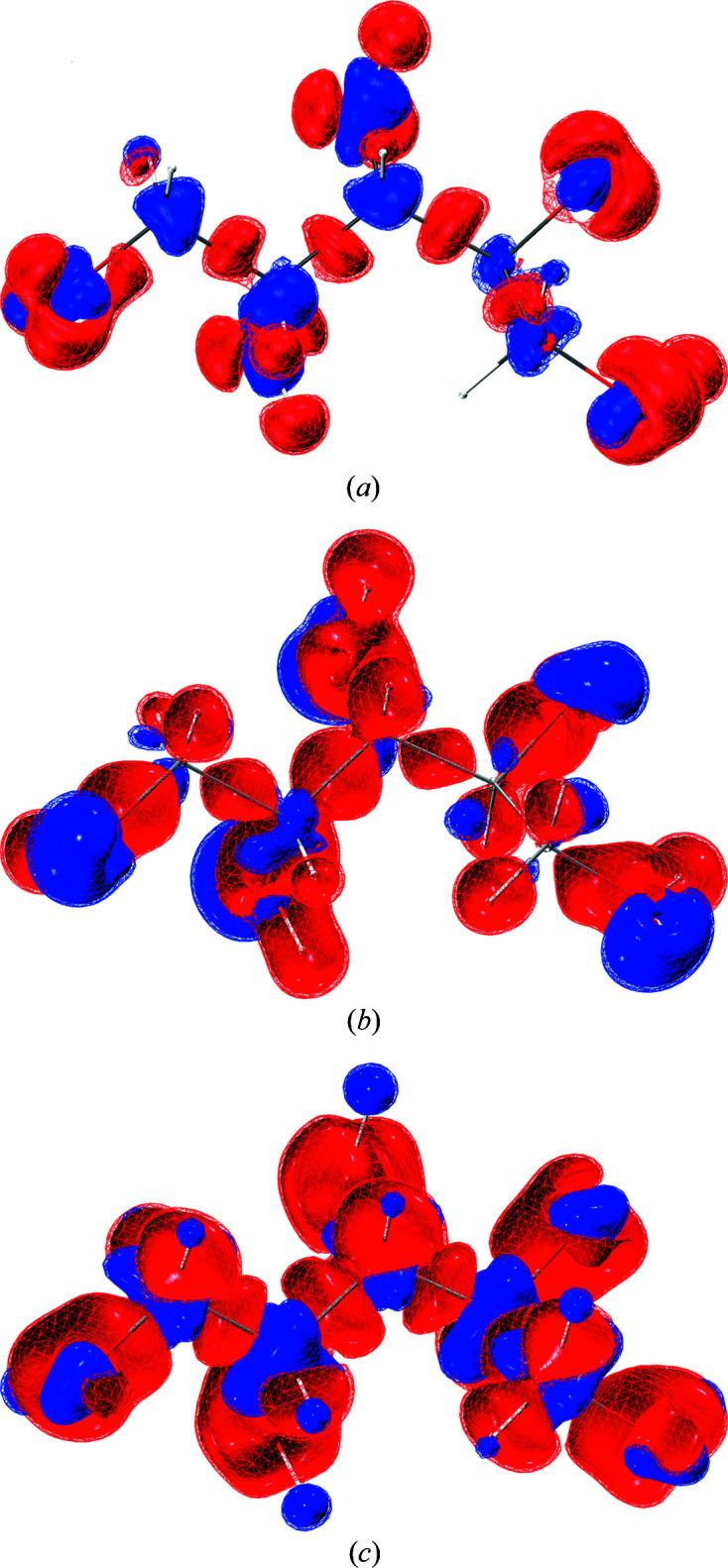
(*a*) Difference of deformation densities visualizing the effect of the XCW fitting for xylitol: deformation density at λ = 1 minus deformation density at λ = 0. (*b*) Difference of deformation densities visualizing the effect of including polarization theoretically: deformation density in a field of Hirshfeld point charges and dipoles minus deformation density in the isolated state (*in vacuo*). (*c*) Difference of deformation densities visualizing the effect of including electron correlation theoretically: deformation density *in vacuo* at the B3LYP level minus deformation density *in vacuo* at the HF level. Isosurfaces: wireframe at 0.025 e Å^−3^ and solid at 0.03 e Å^−3^. Blue = positive, red = negative. Graphic produced with the software *VMD* (Humphrey *et al.*, 1996 ▸). In this case, unlike the previous examples, the differences of deformation densities shown are identical to the total electron density differences since the spherical atomic densities are identical in the two models (at λ = 0 and λ = 1) and, in addition, in both grid files the geometries used to calculate the difference are also identical.

X-ray wavefunction refinement consists of a HAR (results for xylitol visualized in Fig. 6 ▸) and a subsequent XCW fitting in the same geometry using the same fixed ADPs (Woińska *et al.*, 2017 ▸). To visualize the experimental effects incorporated into the molecular wavefunction of xylitol by the XCW fitting procedure, we calculated the deformation density of the model at the XCW λ = 0.0 step [no fitting, purely theoretical unperturbed electron density, χ^2^ = 0.5546, *R*(*F*) = 0.0177, δρ_max_ = 0.126 e Å^−3^] and λ = 1.0 step [after the XCW fitting, experimental information incorporated, χ^2^ = 0.4346, *R*(*F*) = 0.0164, δρ_max_ = 0.116 e Å^−3^]. The difference of the deformation densities is shown in Fig. 7 ▸(*a*). The improvements in the refinement figures of merit χ^2^, *R*(*F*) and δρ_max_ indicate that the difference deformation density features are physically reasonable.

The red colour code in Fig. 7 ▸(*a*) means less electron density in the fitted wavefunction. Red regions can clearly be identified as chemical bonds and lone pairs, whereas blue regions are located around atomic cores. This means that the inseparable combination of electron correlation and polarization via the crystal field leads to a charge redistribution away from the bonding and lone-pair regions towards the atomic cores. This agrees with previous findings for electron correlation (Genoni *et al.*, 2017 ▸), and is also understandable for intermolecular polarization via hydrogen bonding where electron density is withdrawn via a charge-transfer process from the oxygen lone pairs towards the acceptor O—H antibonding orbital. Here, the effect is large: because of the many hydrogen bonds in xylitol, the total number of electrons shifted during the XCW fitting procedure amounts to 3.41 e, obtained via integration of the difference deformation density grid file. For an epoxysuccinyl amide of the same size as xylitol (nine non-H period-2 atoms in epoxysuccinyl amide instead of ten in xylitol), we find that the total number of electrons shifted during the XCW fitting procedure is only 1.9 e (Kleemiss *et al.*, 2021 ▸), thus confirming the impact of the extended hydrogen-bonding network in xylitol on the intermolecular polarization of the molecule.

A theoretical approximation of the individual effects (polarization and correlation) is possible in the following way. Fig. 7 ▸(*b*) depicts the difference deformation density between a wavefunction perturbed by the 8 Å cluster of Hirshfeld point charges and dipoles normally used in HAR and an *in vacuo* wavefunction. This is also called interaction density and measures polarization inside the crystal field (Kleemiss *et al.*, 2021 ▸; Dittrich & Spackman, 2007 ▸; Dittrich *et al.*, 2012 ▸). Electron density is only redistributed in the valence region, from the bonding to the lone-pair density.

Fig. 7 ▸(*c*) depicts the difference deformation density between a wavefunction in the B3LYP approximation, including a certain amount of electron correlation, and an HF wavefunction lacking any treatment of Coulomb correlation. Here, the effect is a redistribution of electron density from the bonds into the core regions of all atoms, including H atoms, as also found for the hydrogen maleate compounds in Section 3.1, and as known in the literature (Wiberg *et al.*, 1992 ▸; Stephens & Becker, 1983 ▸; Genoni *et al.*, 2017 ▸). As described in the previous paragraph, this behaviour is very well mirrored in Fig. 7 ▸(*a*), where the combined effect is fitted via the experimental diffraction data. This means that here we present an experimental verification of the hitherto only theoretically estimated effect of electron correlation on the electron-density distribution of molecules.

It is known that both theoretical approaches overestimate the respective effects significantly. The effect of electron correlation is overestimated by using a hybrid DFT functional (Medvedev *et al.*, 2017 ▸); the effect of polarization is overestimated by using self-consistent Hirshfeld charges and dipoles (Kleemiss *et al.*, 2021 ▸). Therefore, XCW fitting is a reliable and meaningful alternative for describing a chemical redistribution of electron density via intramolecular correlation and intermolecular hydrogen bonding from an experiment. However, it has other shortcomings, such as a dependence on the resolution or on the quality of the experimental data (Genoni *et al.*, 2017 ▸; Ernst *et al.*, 2020 ▸).

## 4. Related literature   

The following literature is cited in the supporting information: Herbst-Irmer & Stalke (2017 ▸); Meindl & Henn (2008 ▸).

## 5. Conclusions and outlook   

The determination of H-atom positions in strong hydrogen bonds by Hirshfeld atom refinement depends on the choice of the QM method and the basis set, with results that vary within about three standard uncertainties. Therefore, it is not surprising that the accurate determination of hydrogen-bonding parameters through HAR-ELMO is influenced by the extremely localized molecular orbital approximation to about the same extent. In xylitol, hydrogen bonding is abundant and is the main cause of a shift of about 3 e between the isolated and the X-ray constrained wavefunction. Although overall HAR seems to be suitable for the determination of H-atom positions even in strong hydrogen bonds as an alternative to neutron-diffraction experiments, it is not able to accurately refine hydrogen anisotropic displacement parameters in strong hydrogen bonds. Hence, quantum-crystallographic modelling and the accuracy of the determination of hydrogen-bonding parameters remain important subjects for further method development and methodological investigations.

For this purpose, we have started investigating the differences between quantum-mechanical electron densities corresponding to the approximate models used in the refinement. It becomes clear that the inclusion of electron correlation effects into the ansatz for HAR is important if one wants to obtain more accurate hydrogen-bonding parameters. However, this needs to be counterbalanced against the speed of the refinements. The loss of accuracy in HAR-ELMO is small, but the speed is significantly higher than in regular HARs, a fact that becomes important for applications in protein crystallography which are currently under investigation. Since we have shown that XCW fitting reliably and correctly incorporates electron correlation and polarization effects into the wavefunction, X-ray constrained applications might be a viable alternative to the inclusion of electron correlation into the wavefunction ansatz for HARs – a method also being developed in our groups to extend the present implementation of X-ray wavefunction refinement.

## Supplementary Material

Crystal structure: contains datablock(s) global, I. DOI: 10.1107/S1600576721001126/in5039sup1.cif

Structure factors: contains datablock(s) Xylitol_XWR. DOI: 10.1107/S1600576721001126/in5039Isup2.hkl

Supporting figures. DOI: 10.1107/S1600576721001126/in5039sup3.pdf

CIFs, FCO, FCF and checkcif report for AGA HAR and HAR-ELMO. DOI: 10.1107/S1600576721001126/in5039sup4.zip

CIFs, FCO, FCF and checkcif report for all hydrogen maleate refinements in all models. DOI: 10.1107/S1600576721001126/in5039sup5.zip

CCDC references: 1987762, 1987825, 1987828, 1987830, 2060247

## Figures and Tables

**Figure 3:**
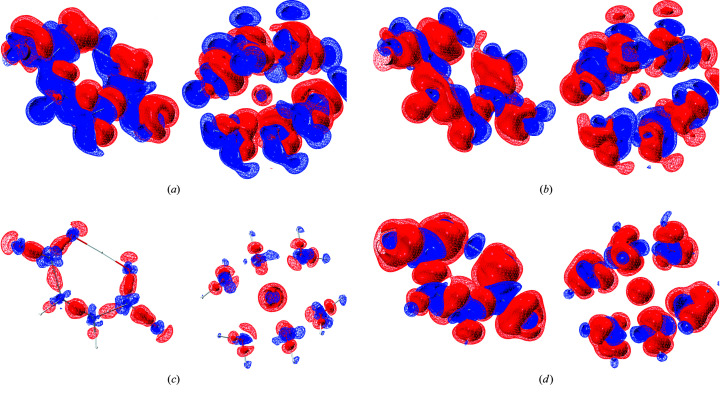
Differences of the theoretical deformation densities underlying Mg HMal refinements at different levels of theory (blue = positive, red = negative; isosurfaces, wireframe at 0.025 e Å^−3^ and solid at 0.05 e Å^−3^). (*a*) B3LYP/def2-TZVP minus HF/def2-TZVP, depicting the effect of electron correlation; (*b*) B3PW91/def2-TZVP minus B3LYP/def2-TZVP, depicting the effect of different hybrid DFT functionals; (*c*) B3PW91/6-311++G(*d*,*p*) minus B3PW91/def2-TZVP, depicting the basis-set dependency; (*d*) B3PW91/6-311++G(*d*,*p*) minus HF/def2-TZVP, depicting the superposition of all effects. The grid files containing the individual deformation density distributions are based on the final geometries after refinement and thus they slightly deviate from each other. Therefore, the difference deformation densities shown here are not exactly identical to the corresponding difference electron densities, but they are qualitatively very similar. The molecular structures shown are always those of the first method mentioned in the differences. Graphics were produced with the program *VMD* (Humphrey *et al.*, 1996 ▸).

**Table 1:** Crystallographic and measurement details (part I)

Compound	8-Hydroxyquinolinium hydrogen maleate	Magnesium bis(hydrogen maleate) hexahydrate
Chemical formula	(C_9_H_8_NO)(C_4_H_3_O_4_)	2(C_4_H_3_O_4_)Mg(H_2_O)_6_
Formula weight (g mol^−1^)	261.24	362.54
Crystal size (mm^3^)	0.119 × 0.092 × 0.066	0.150 × 0.130 × 0.100
Crystal habit	Needle	Block
Crystal colour	Yellow	Colourless
Temperature (K)	15 (2)	14.9 (2)
Wavelength (Å)	0.35307	0.3532

Unit cell		
*a* (Å)	5.33860 (10)	10.195 (2)
*b* (Å)	9.9878 (2)	11.759 (2)
*c* (Å)	22.3493 (4)	6.6206 (13)
α (°)	90.00	90.00
β (°)	90.00	103.67 (3)
γ (°)	90.00	90.00
Volume (Å^3^)	1191.68 (4)	771.2 (3)
*Z*	4	2
Space group	*P*2_1_2_1_2_1_	*P*2_1_/*c*

No. of reflections	80 044	155 955
*R*_int_/completeness/redundancy	0.0522/99.6%/5.12	0.0265/99.7%/12.39
Unique reflections	15 641	12 589
Unique observed [*F*/σ(*F*) > 4]	12 569	11 646
Reflections θ_min_ (°)	0.91	1.02
Reflections θ_max_ (°)	24.24 (*d* = 0.43 Å)	26.20 (*d* = 0.40 Å)

**Table 2:** Crystallographic and measurement details (part II)

Compound	L-Alanyl-glycyl-L-alanine	Xylitol
Chemical formula	C_8_H_15_N_3_O_4_·H_2_O	C_5_H_12_O_5_
Formula weight (g mol^−1^)	235.2418	152.1484
Crystal size (mm^3^)	0.350 × 0.300 × 0.250	0.370 × 0.320 × 0.260
Crystal habit	Needle	Prism
Crystal colour	Colourless	Colourless
Temperature (K)	92 (2)	122.4 (5)
Wavelength (Å)	0.6214	0.71073

Unit cell		
*a* (Å)	10.224 (6)	8.264 (4)
*b* (Å)	4.804 (3)	8.901 (2)
*c* (Å)	11.987 (7)	8.9223 (14)
α (°)	90.00	90.00
β (°)	101.419 (13)	90.00
γ (°)	90.00	90.00
Volume (Å^3^)	577.1 (6)	656.3 (4)
*Z*	2	4
Space group	*P*2_1_	*P*2_1_2_1_2_1_

No. of reflections	28 133	33 102
*R*_int_/completeness/redundancy	0.0302/90.3%/2.99	0.0317/100%/3.33
Unique reflections	9406	9942
Unique observed [*F*/σ(*F*) > 4]	8658	8894
Reflections θ_min_ (°)	1.52	3.23
Reflections θ_max_ (°)	50.70 (*d* = 0.40 Å)	59.94 (*d* = 0.41 Å)

**Table:** HAR performed at four different levels of theory for each compound. The neutron-diffraction-derived distances are 1.072 (3) and 1.378 (4) Å for 8HQ HMal and 1.1873 (16) and 1.2181 (16) Å for Mg HMal.

8HQ HMal	HF/def2-TZVP	B3LYP/def2-TZVP	B3PW91/def2-TZVP	B3PW91/6-311++G(*d*,*p*)
*R*[*F* > 4σ(*F*)]	0.033	0.033	0.033	0.033
*wR*(*F*)	0.020	0.019	0.019	0.020
χ^2^	2.757	2.527	2.500	2.596
δρ_max_ (e Å^−3^)	0.309	0.331	0.328	0.348
δρ_min_ (e Å^−3^)	−0.301	−0.289	−0.285	−0.286
δρ_mean_ (e Å^−3^)	0.047	0.047	0.047	0.047
*d*(O1—H1) (Å)	1.083 (6)	1.042 (8)	1.043 (8)	1.049 (8)
*d*(O2—H1) (Å)	1.369 (6)	1.403 (8)	1.402 (8)	1.396 (8)

**Table:** 

Mg HMal	HF/def2-TZVP	B3LYP/def2-TZVP	B3PW91/def2-TZVP	B3PW91/6-311++G(*d*,*p*)
*R*[*F* > 4σ(*F*)]	0.016	0.016	0.016	0.016
*wR*(*F*)	0.017	0.016	0.016	0.016
χ^2^	9.094	8.410	8.116	8.712
δρ_max_ (e Å^−3^)	0.401	0.409	0.407	0.406
δρ_min_ (e Å^−3^)	−0.324	−0.322	−0.318	−0.315
δρ_mean_ (e Å^−3^)	0.026	0.027	0.026	0.027
*d*(O1—H1) (Å)	1.183 (4)	1.189 (4)	1.192 (4)	1.195 (4)
*d*(O2—H1) (Å)	1.224 (4)	1.217 (4)	1.214 (4)	1.211 (4)

**Table 4:** Statistics for the HAR and HAR-ELMO treatment of AGA The time is the wall-clock time on a single CPU.

	HAR	HAR-ELMO
*R*[*F* > 4σ(*F*)]	0.033	0.033
*wR*(*F*)	0.024	0.025
χ^2^	5.805	6.143
δρ_max_ (e Å^−3^)	0.237	0.245
δρ_min_ (e Å^−3^)	− 0.244	− 0.238
δρ_mean_ (e Å^−3^)	0.037	0.038
Time	52 min 37 s	12 min 18 s
