# Supplementary material for: The advanced treatment of hydrogen bonding in quantum crystallography
Source: J Appl Crystallogr. 2021 Apr 16;54(Pt 3):718–29. doi: 10.1107/S1600576721001126 (PMC8202034; doi:10.1107/S1600576721001126)
Supplement: Supplementary file 3 [file j-54-00718-sup3.pdf]

JOURNAL OF APPLIED CRYSTALLOGRAPHY

Volume 54 (2021)

Supporting information for article:

The advanced treatment of hydrogen bonding in quantum crystallography

Lorraine A. Malaspina, Alessandro Genoni, Dylan Jayatilaka, Michael J. Turner, Kunihisa Sugimoto, Eiji Nishibori and Simon Grabowsky

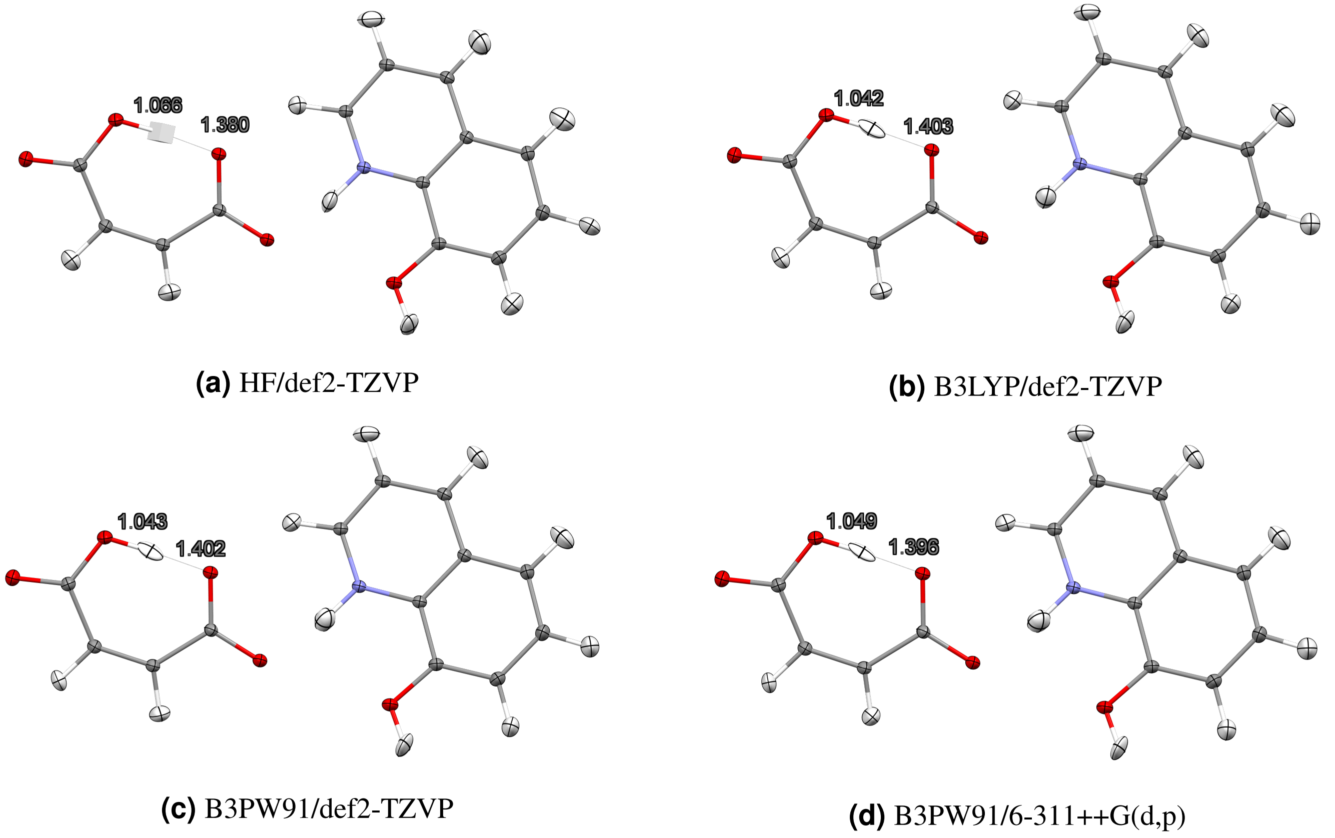

(a) HF/def2-TZVP

(b) B3LYP/def2-TZVP

(c) B3PW91/def2-TZVP

(d) B3PW91/6-311++G(d,p)

Figure S1. Molecular structures of 8HQ HMal after HARs at four different levels of theory. Distances are given in Å. ADPs are given at 50% probability level.

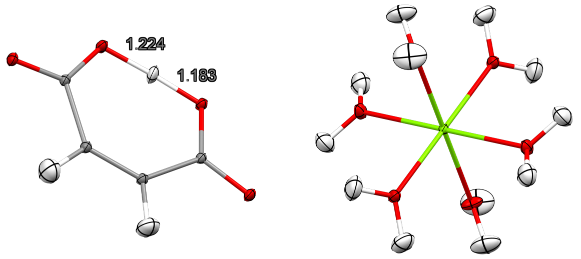

(a) HF/def2-TZVP

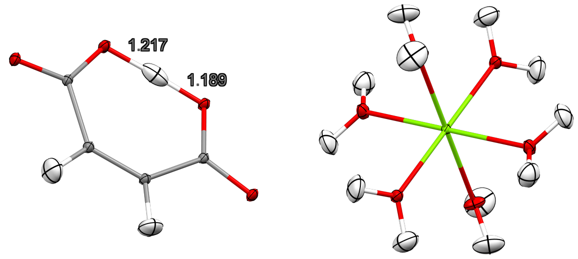

(b) B3LYP/def2-TZVP

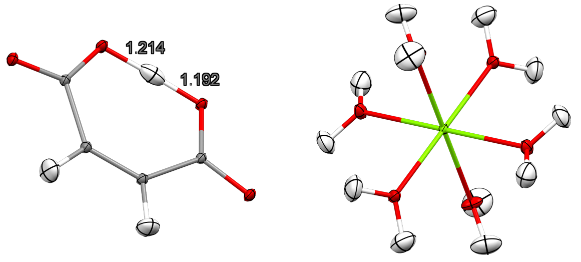

(c) B3PW91/def2-TZVP

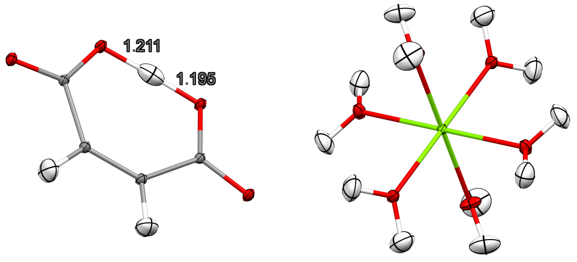

(d) B3PW91/6-311++G(d,p)

Figure S2. Molecular structures of Mg HMal after HARs at four different levels of theory. Distances are given in Å. ADPs are given at 50% probability level.

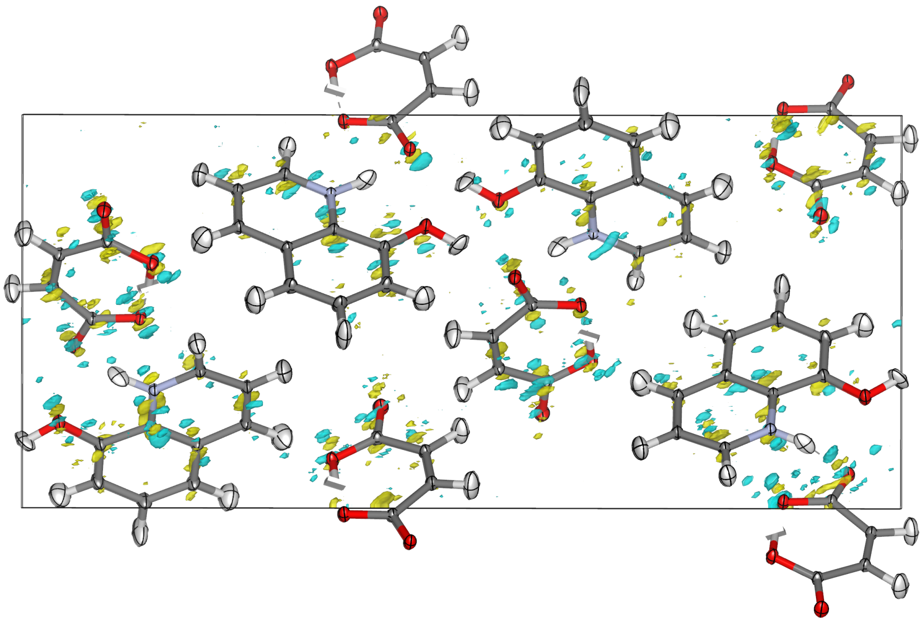

(a) HF/def2-TZVP

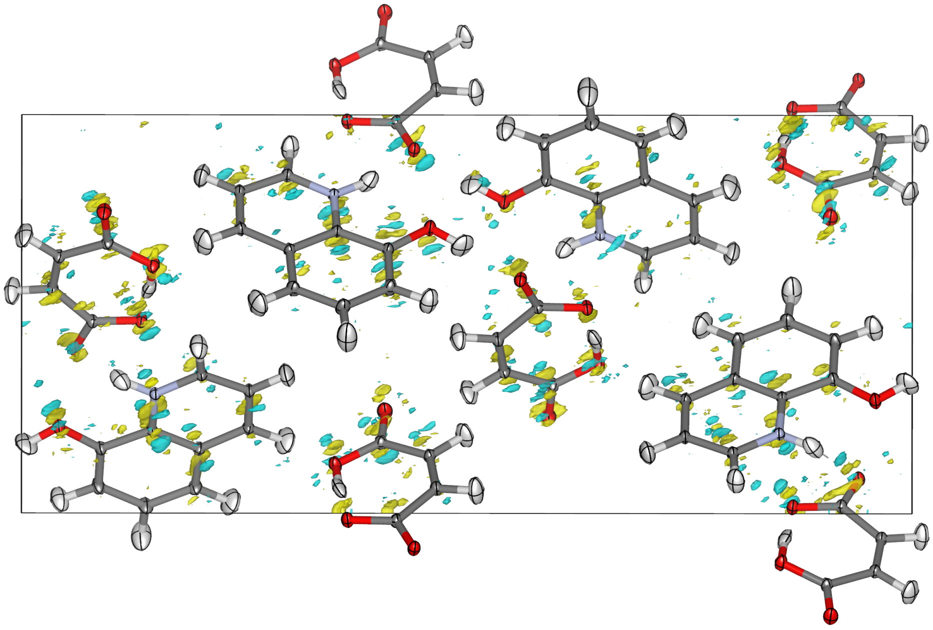

(b) B3LYP/def2-TZVP

Figure S3. Residual electron density distributions of 8HQ HMal after HARs at two different levels of theory. Yellow=positive, light-blue=negative. Isovalues are ± 0.10 e/Å^3.

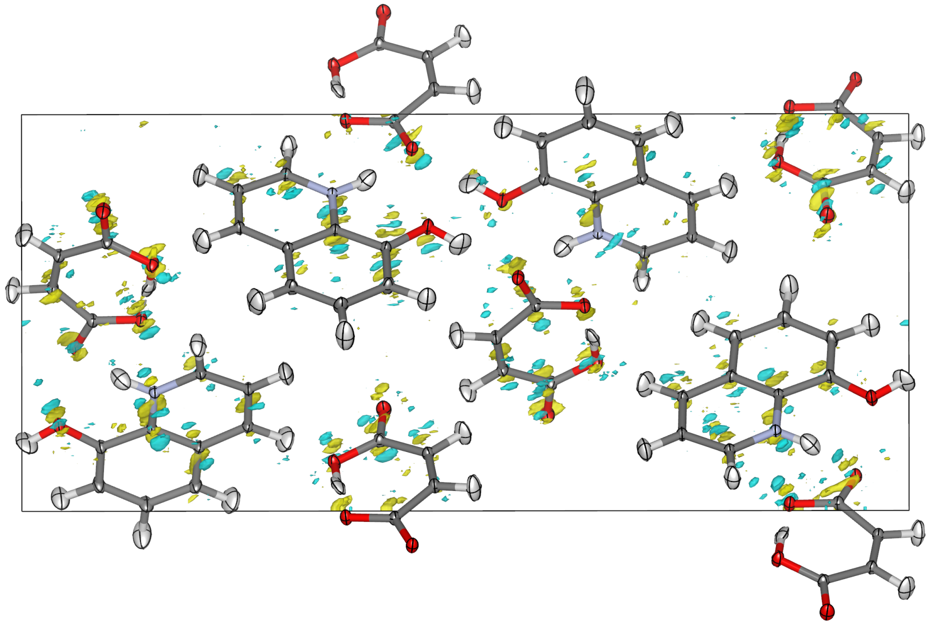

(a) B3PW91/def2-TZVP

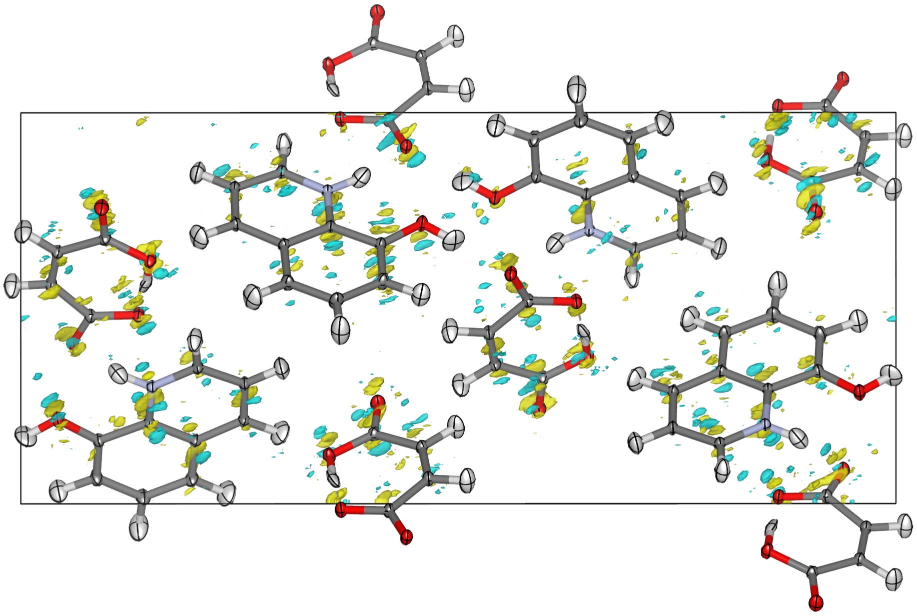

(b) B3PW91/6-311++G(d,p)

Figure S4. Residual electron density distributions of 8HQ HMal after HARs at two different levels of theory. Yellow=positive, light-blue=negative. Isovalues are ± 0.15 e/Å^3.

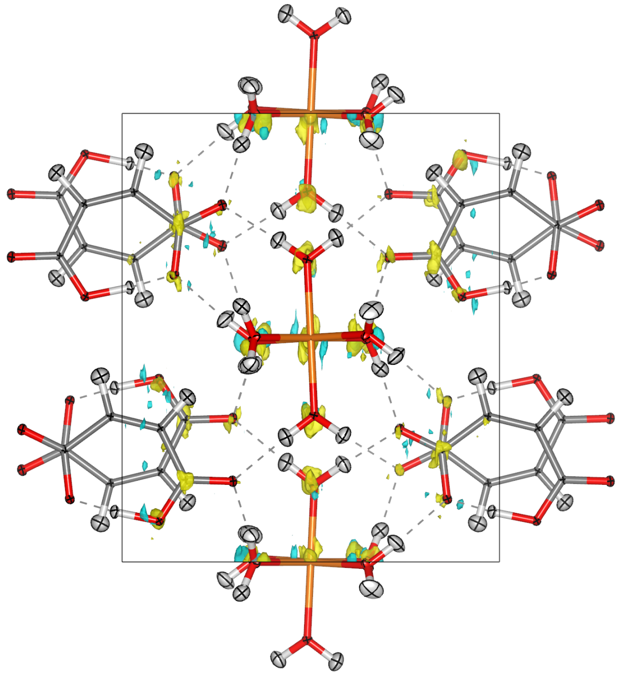

(a) HF/def2-TZVP

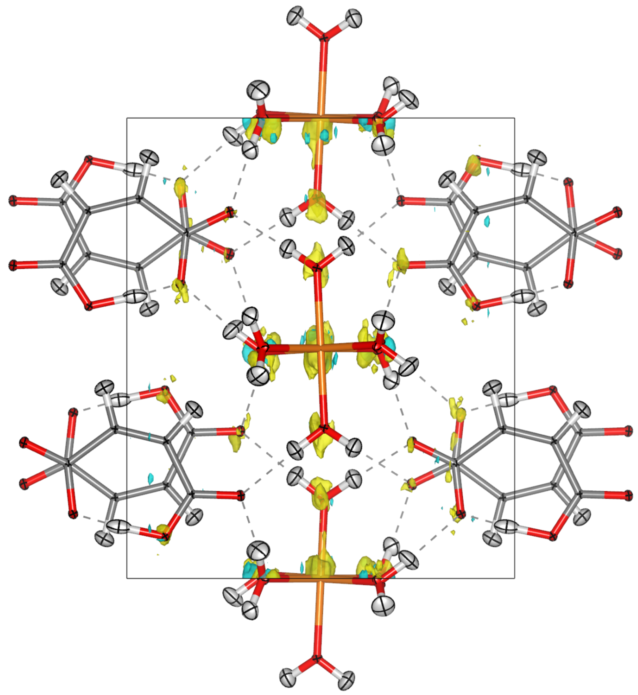

(b) B3LYP/def2-TZVP

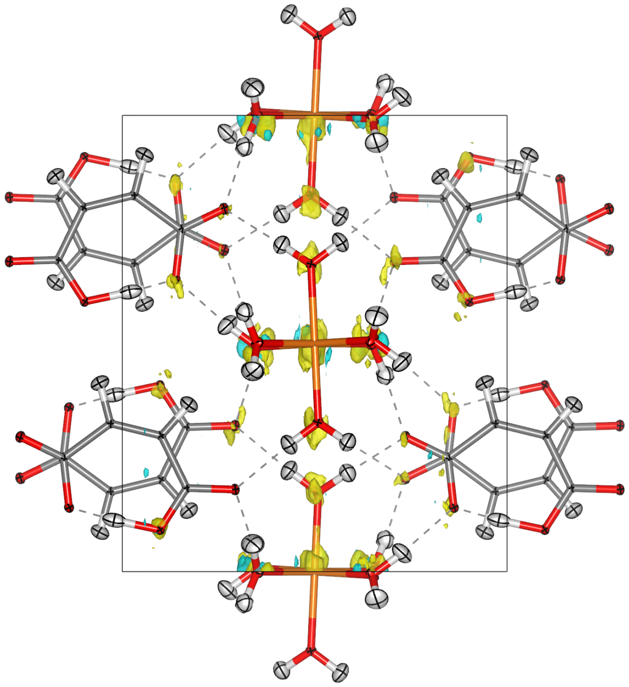

(c) B3PW91/def2-TZVP

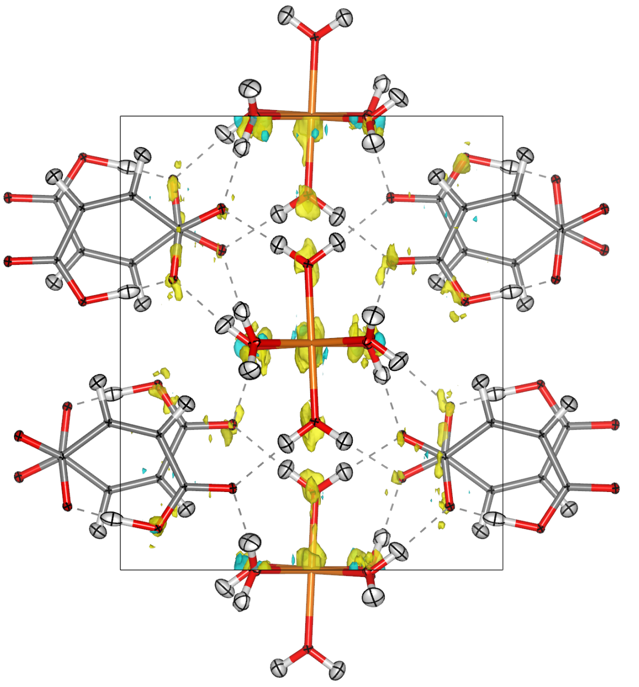

(d) B3PW91/6-311++G(d,p)

Figure S5. Residual electron density distributions of 8HQ HMal after HARs at four different levels of theory. Yellow=positive, light-blue=negative. Isovalues are $\pm$ 0.15 e/Å^3.

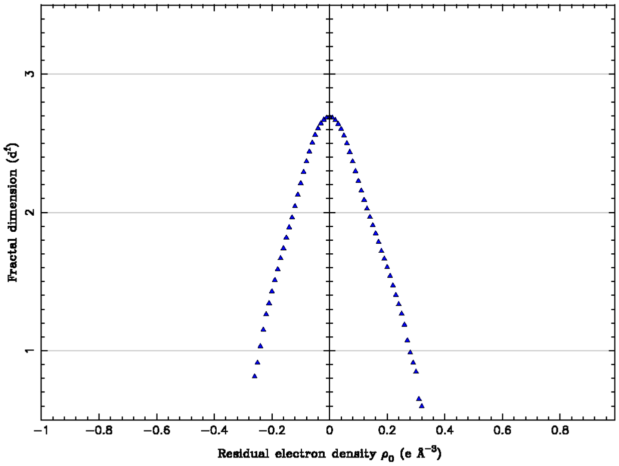

(a) 8HQ HM *Gaussian*-HAR

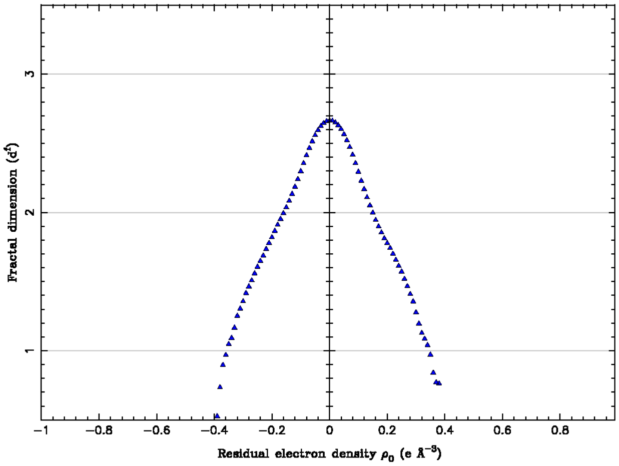

(b) 8HQ HM regular HAR

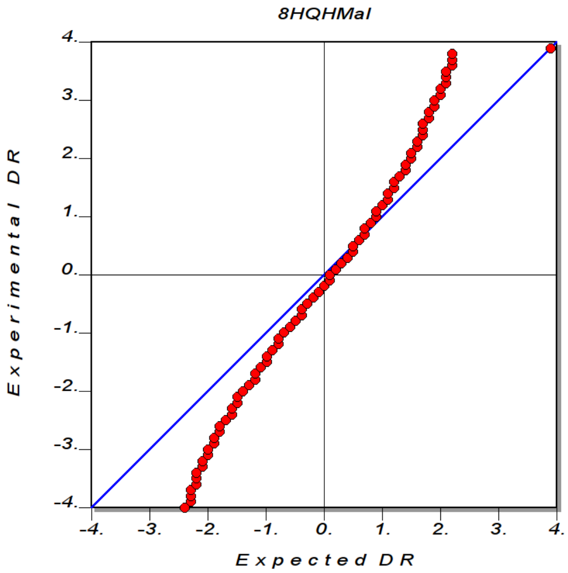

(c) 8HQ HM *Gaussian*-HAR

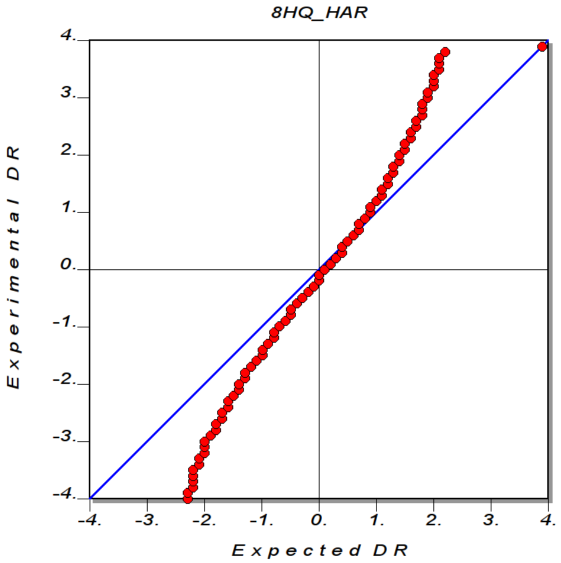

(d) 8HQ HM regular HAR

Figure S6. Comparison between the *Gaussian*-HAR refinement at the B3PW91/6-311++G(d,p) level of theory and the regular HAR at the HF/def2-TZVP level of theory for 8HQ HM. First row: Fractal dimension plots of the residual electron density according to Meindl and Henn [1]. Second row: Normal probability plots.

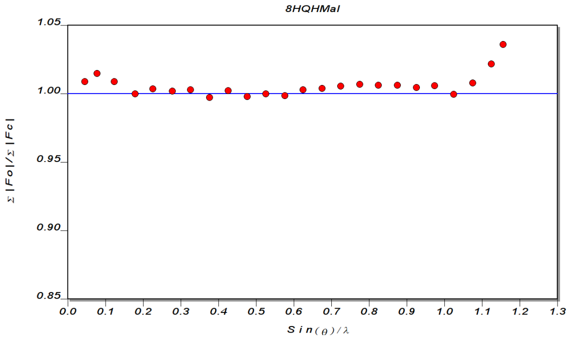

(a) 8HQ HM *Gaussian*-HAR

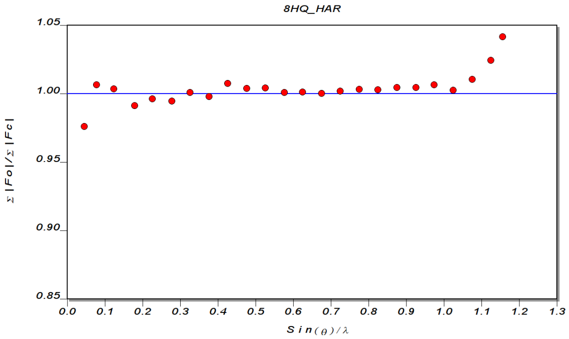

(b) 8HQ HM regular HAR

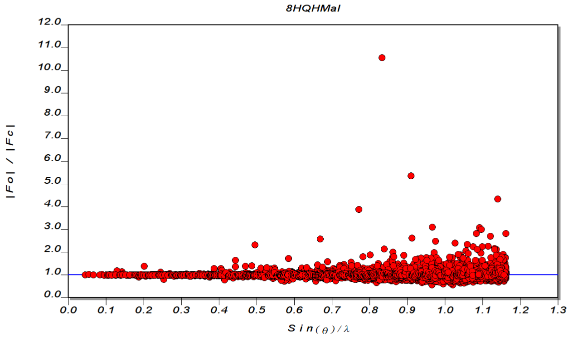

(c) 8HQ HM *Gaussian*-HAR

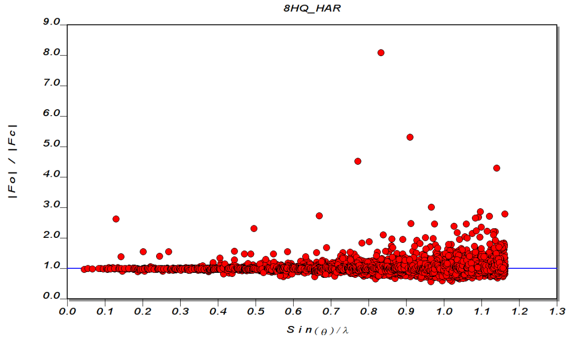

(d) 8HQ HM regular HAR

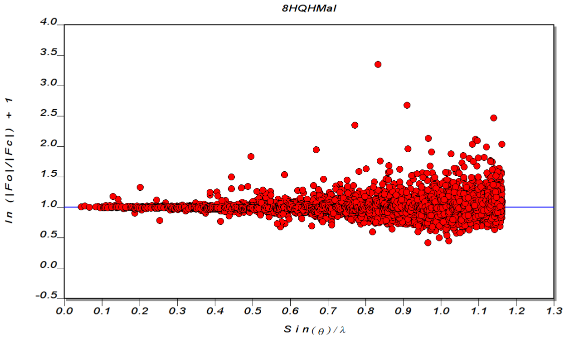

(e) 8HQ HM *Gaussian*-HAR

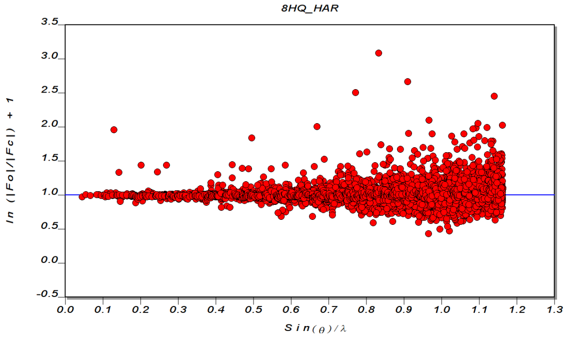

(f) 8HQ HM regular HAR

Figure S7. Comparison between the *Gaussian*-HAR refinement at the B3PW91/6-311++G(d,p) level of theory and the regular HAR at the HF/def2-TZVP level of theory for 8HQ HM. First row: DRK-plots [2]. Second and third rows: Scatter plots.

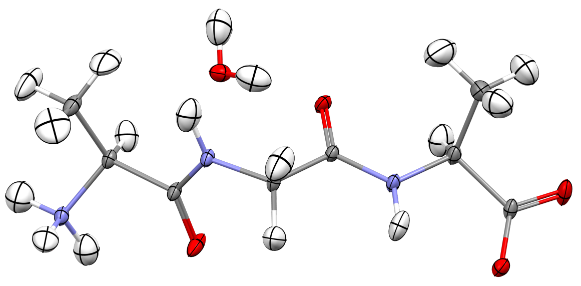

(a) AGA HAR-ELMO

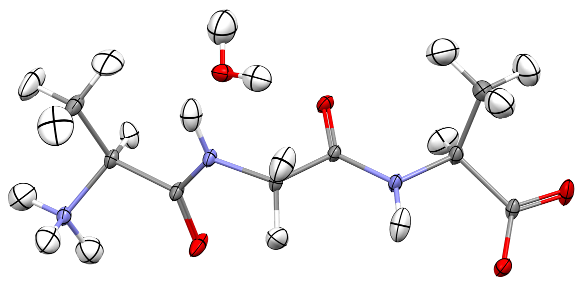

(b) AGA HAR

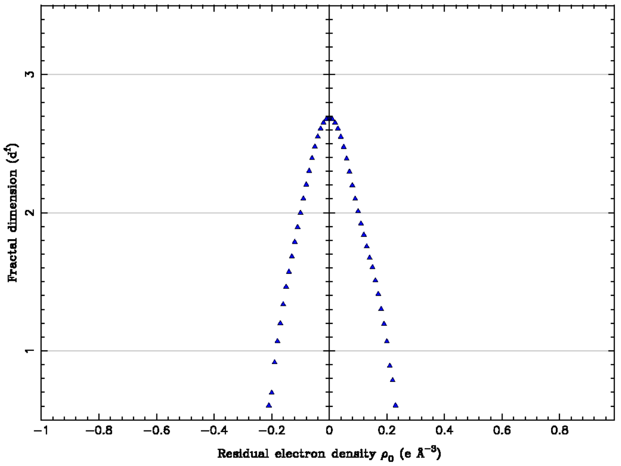

(c) AGA HAR-ELMO

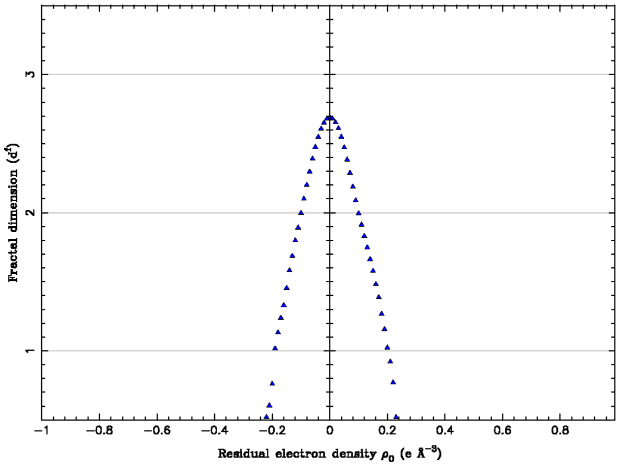

(d) AGA HAR

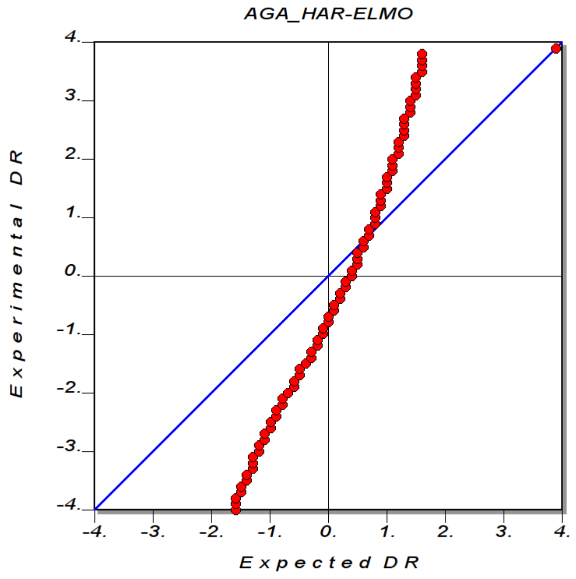

(e) AGA HAR-ELMO

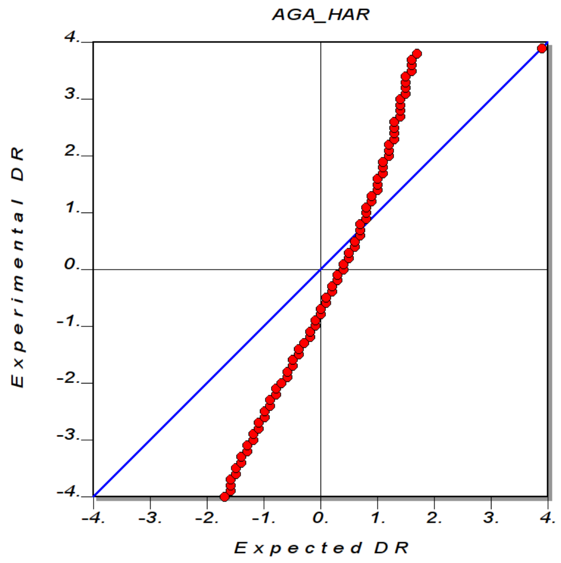

(f) AGA HAR

Figure S8. Comparison between the HAR-ELMO refinement and the regular HAR of *L*-Ala-Gly-*L*-Ala. First row: Molecular structures with refined ADPs at 50% probability. Second row: Fractal dimension plots of the residual electron density according to Meindl and Henn [1]. Third row: Normal probability plots.

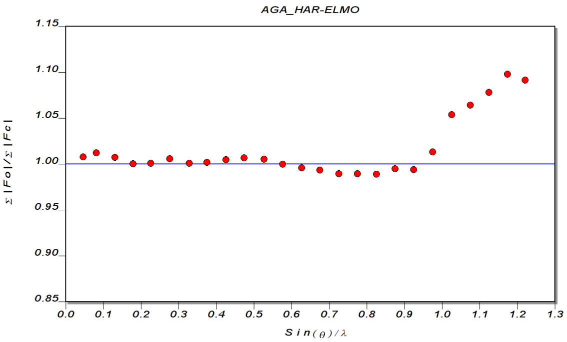

(a) AGA HAR-ELMO

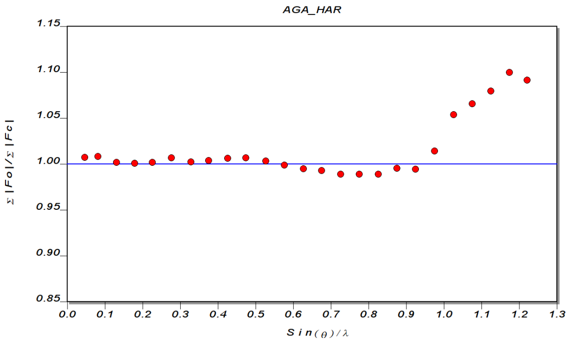

(b) AGA HAR

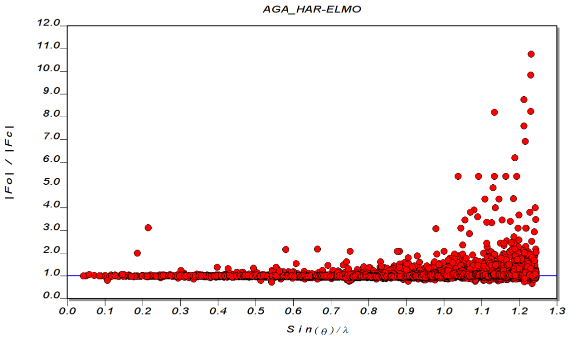

(c) AGA HAR-ELMO

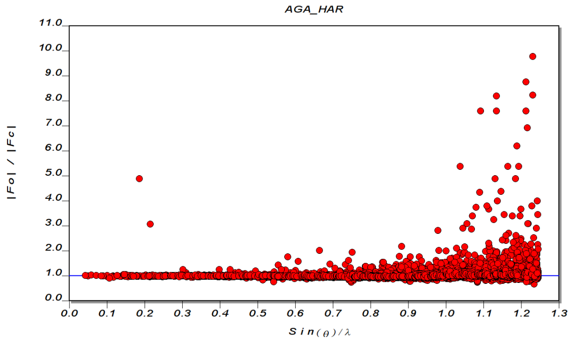

(d) AGA HAR

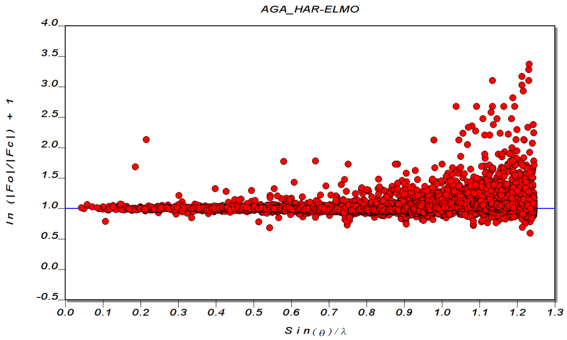

(e) AGA HAR-ELMO

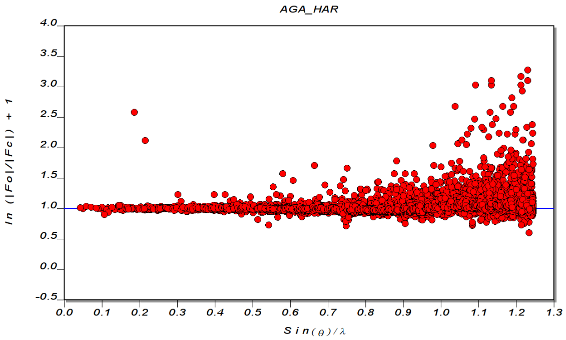

(f) AGA HAR

Figure S9. Comparison between the HAR-ELMO refinement and the regular HAR of *L*-Ala-Gly-*L*-Ala. First row: DRK-plots [2]. Second and third rows: Scatter plots.

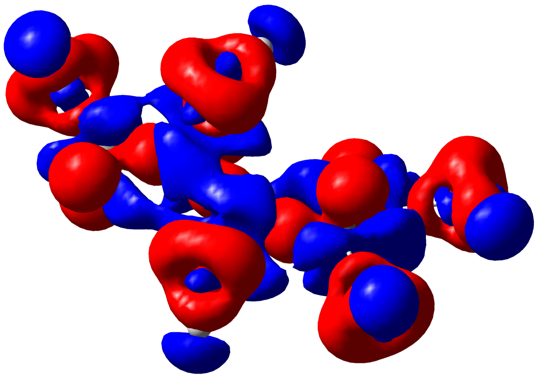

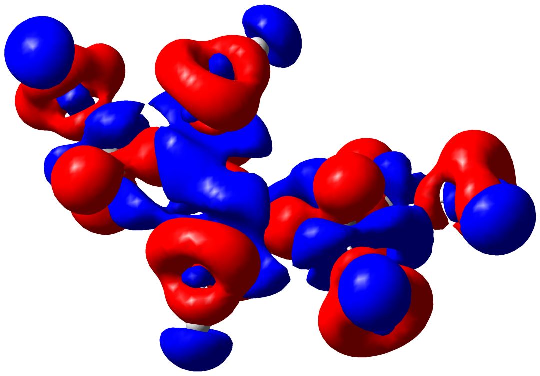

(a) Xylitol after HAR **(b)** Xylitol after XCW fitting

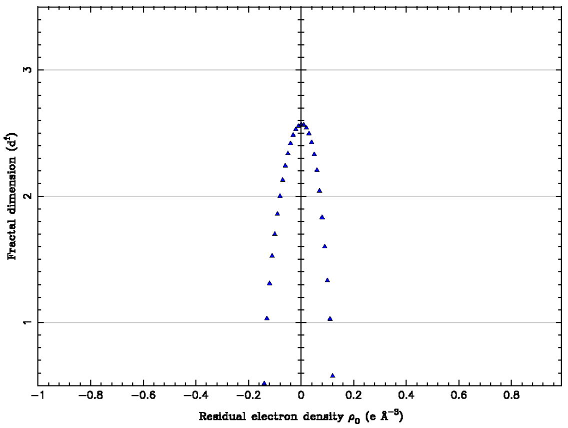

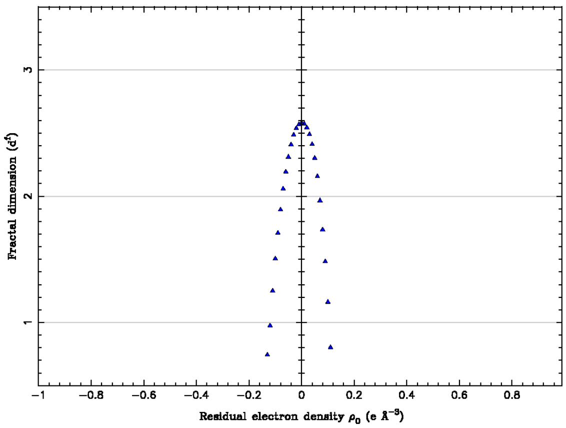

(c) Xylitol after HAR **(d)** Xylitol after XCW fitting

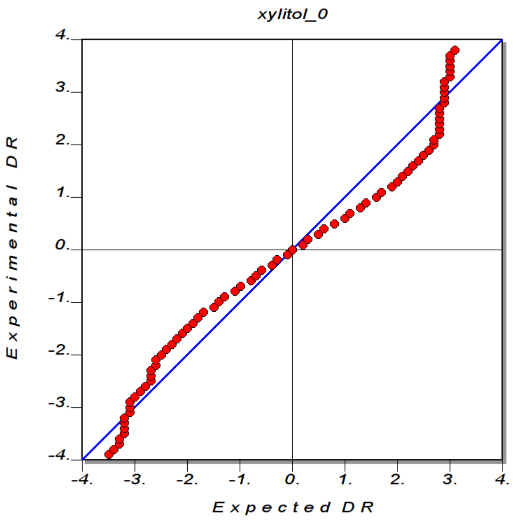

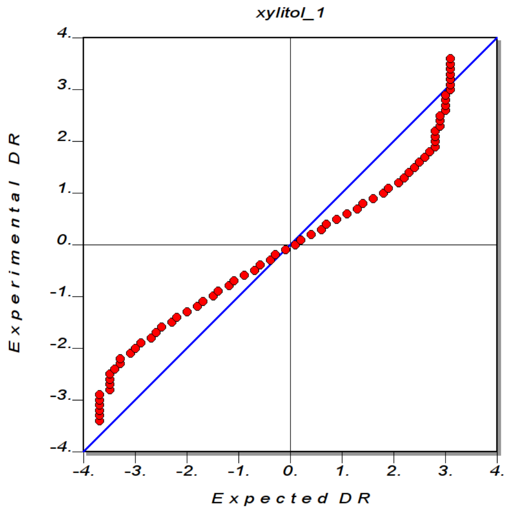

(e) Xylitol after HAR **(f)** Xylitol after XCW fitting

Figure S10. Comparison between the HAR refinement (HF/6-311G(d,p) with cluster charges and dipoles) and XCW fitting (HF/6-311G(d,p) without cluster charges and dipoles) models of xylitol. First row: Deformation density map at an isovalue of $\pm$ 0.08 eÅ^{-3}, red = positive, blue = negative. Second row: Fractal dimension plots of the residual electron density according to Meindl and Henn [1]. Third row: Normal probability plots.

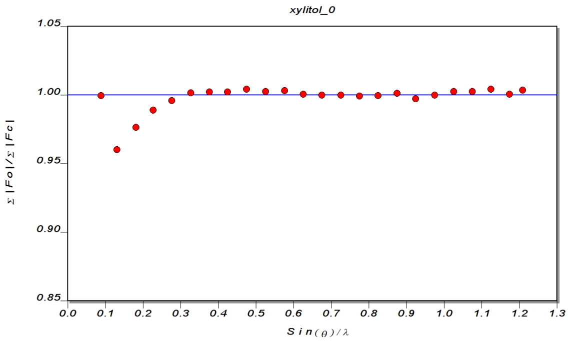

(a) Xylitol after HAR

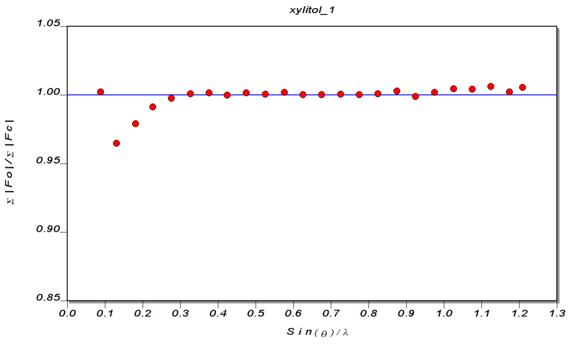

(b) Xylitol after XCW fitting

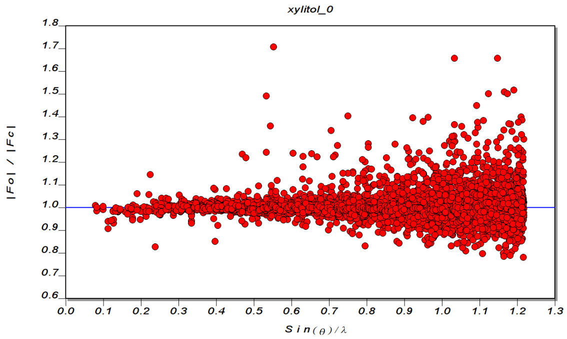

(c) Xylitol after HAR

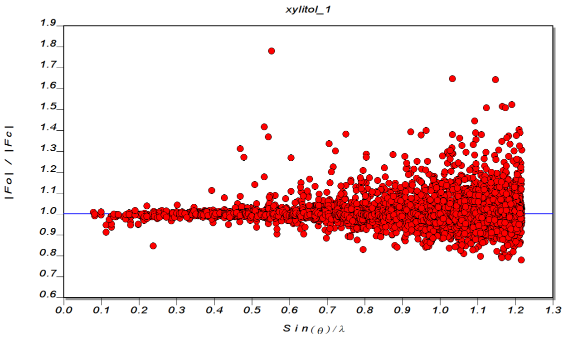

(d) Xylitol after XCW fitting

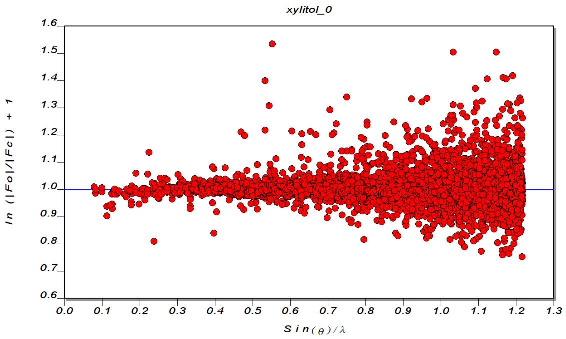

(e) Xylitol after HAR

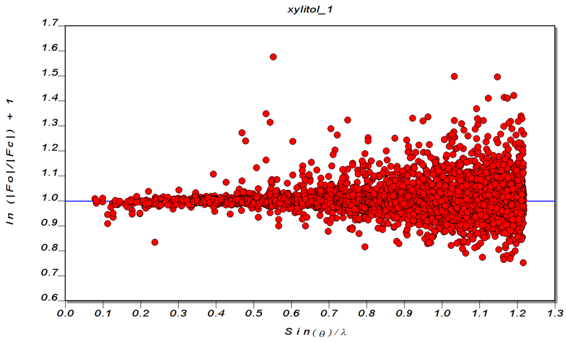

(f) Xylitol after XCW fitting

Figure S11. Comparison between the HAR refinement and XCW fitting models of xylitol. First row: DRK-plots [2]. Second and third rows: Scatter plots.

References

1. K. Meindl and J. Henn, *Acta Cryst. A*, 2008, **64**, 404–418.

2. R. Herbst-Irmer and D. Stalke, *Acta Cryst. B*, 2017, **73**, 531–543.
