# Supplementary material for: The advanced treatment of hydrogen bonding in quantum crystallography
Source: J Appl Crystallogr. 2021 Apr 16;54(Pt 3):718–29. doi: 10.1107/S1600576721001126 (PMC8202034; doi:10.1107/S1600576721001126)

# checkCIF/PLATON report

Structure factors have been supplied for datablock(s) aga_har-elmo

THIS REPORT IS FOR GUIDANCE ONLY. IF USED AS PART OF A REVIEW PROCEDURE FOR PUBLICATION, IT SHOULD NOT REPLACE THE EXPERTISE OF AN EXPERIENCED CRYSTALLOGRAPHIC REFEREE.

No syntax errors found.    CIF dictionary    Interpreting this report

# Datablock: aga_har-elmo

---

```
Bond precision:  C-C = 0.0014 A          Wavelength=0.62140

Cell:            a=10.224(6)     b=4.804(3)            c=11.987(7)
                 alpha=90        beta=101.419(13)      gamma=90
Temperature:     92 K

                Calculated                 Reported
Volume          577.1(6)                   577.1(6)
Space group     P 21                       P 1 21 1
Hall group      P 2yb                      P 2yb
Moiety formula  C8 H15 N3 O4, H2 O         C8 H15 N3 O4, H2 O
Sum formula     C8 H17 N3 O5               C8 H17 N3 O5
Mr              235.25                     235.24
Dx,g cm-3       1.354                      1.354
Z               2                          2
Mu (mm-1)       0.083                      0.112
F000            252.0                      252.0
F000'           252.08
h,k,lmax        25,11,29                   25,10,29
Nref            18638[ 9904]               9406
Tmin,Tmax       0.962,0.972
Tmin'           0.962

Correction method= Not given

Data completeness= 0.95/0.50      Theta(max)= 50.695

R(reflections)= 0.0331( 8658)     wR2(reflections)= wR= 0.0250(
                                  8658)

S = 1.020                    Npar= 297
```

---

The following ALERTS were generated. Each ALERT has the format
**test-name_ALERT_alert-type_alert-level**.
Click on the hyperlinks for more details of the test.

---

## Alert level A

```
PLAT183_ALERT_1_A Missing _cell_measurement_reflns_used Value ....     Please Do !
```

**Author Response: This is a re-refinement of deposited data CCDC 627276 (TAPYEP02). These values were not reported by the original authors.**

```
PLAT184_ALERT_1_A Missing _cell_measurement_theta_min Value ......     Please Do !
```

**Author Response: This is a re-refinement of deposited data CCDC 627276 (TAPYEP02). These values were not reported by the original authors.**

```
PLAT185_ALERT_1_A Missing _cell_measurement_theta_max Value ......     Please Do !
```

**Author Response: This is a re-refinement of deposited data CCDC 627276 (TAPYEP02). These values were not reported by the original authors.**

---

## Alert level C

```
REFLE01_ALERT_3_C  The _reflns_threshold_multiplier given is >= 4
            Premultiplier =    4.01
REFLE01_ALERT_3_C  The _reflns_threshold_multiplier given is >= 4
            Premultiplier =    4.01
PLAT029_ALERT_3_C _diffrn_measured_fraction_theta_full value Low .      0.976 Why?
PLAT351_ALERT_3_C Long   C-H (X0.96,N1.08A)  C1       - H1       .       1.11 Ang.
PLAT351_ALERT_3_C Long   C-H (X0.96,N1.08A)  C8       - H8B      .       1.12 Ang.
PLAT353_ALERT_3_C Long   N-H (N0.87,N1.01A)  N1       - H11A     .       1.02 Ang.
PLAT353_ALERT_3_C Long   N-H (N0.87,N1.01A)  N1       - H11B     .       1.01 Ang.
PLAT353_ALERT_3_C Long   N-H (N0.87,N1.01A)  N1       - H11C     .       1.04 Ang.
PLAT353_ALERT_3_C Long   N-H (N0.87,N1.01A)  N3       - H13      .       1.01 Ang.
PLAT975_ALERT_2_C Check Calcd Resid. Dens.  0.49A   From N1              0.44 eA-3
PLAT975_ALERT_2_C Check Calcd Resid. Dens.  0.58A   From N1              0.44 eA-3
PLAT975_ALERT_2_C Check Calcd Resid. Dens.  0.44A   From O5              0.40 eA-3
PLAT978_ALERT_2_C Number C-C Bonds with Positive Residual Density.          0 Info
```

---

## Alert level G

```
ABSMU01_ALERT_1_G  Calculation of _exptl_absorpt_correction_mu
                not performed for this radiation type.
PLAT032_ALERT_4_G Std. Uncertainty on Flack Parameter Value High .      0.400 Report
PLAT092_ALERT_4_G Check: Wavelength Given is not Cu,Ga,Mo,Ag,In Ka    0.62140 Ang.
PLAT791_ALERT_4_G Model has Chirality at C1          (Chiral SPGR)          S Verify
PLAT791_ALERT_4_G Model has Chirality at C5          (Chiral SPGR)          S Verify
PLAT808_ALERT_5_G No Parseable SHELXL Style Weighting Scheme Found     Please Check
PLAT881_ALERT_1_G No Datum for _diffrn_reflns_av_R_equivalents ...     Please Do !
PLAT911_ALERT_3_G Missing FCF Refl Between Thmin & STh/L=    0.600         10 Report
PLAT912_ALERT_4_G Missing # of FCF Reflections Above STh/L=  0.600       1058 Note
PLAT929_ALERT_5_G No Weight Pars,Obs and Calc R1,wR2,S not Checked          ! Info
PLAT961_ALERT_5_G Dataset Contains no Negative Intensities .......     Please Check
PLAT981_ALERT_1_G No non-zero f" Anomalous Scattering Values Found     Please Check
PLAT986_ALERT_1_G No non-zero f' Anomalous Scattering Values Found     Please Check
```

```
PLAT992_ALERT_5_G Repd & Actual _reflns_number_gt Values Differ by          3 Check
```

---

```
  3 ALERT level A = Most likely a serious problem - resolve or explain
  0 ALERT level B = A potentially serious problem, consider carefully
 13 ALERT level C = Check. Ensure it is not caused by an omission or oversight
 14 ALERT level G = General information/check it is not something unexpected

  7 ALERT type 1 CIF construction/syntax error, inconsistent or missing data
  4 ALERT type 2 Indicator that the structure model may be wrong or deficient
 10 ALERT type 3 Indicator that the structure quality may be low
  5 ALERT type 4 Improvement, methodology, query or suggestion
  4 ALERT type 5 Informative message, check
```

---

It is advisable to attempt to resolve as many as possible of the alerts in all categories. Often the minor alerts point to easily fixed oversights, errors and omissions in your CIF or refinement strategy, so attention to these fine details can be worthwhile. In order to resolve some of the more serious problems it may be necessary to carry out additional measurements or structure refinements. However, the purpose of your study may justify the reported deviations and the more serious of these should normally be commented upon in the discussion or experimental section of a paper or in the "special_details" fields of the CIF. checkCIF was carefully designed to identify outliers and unusual parameters, but every test has its limitations and alerts that are not important in a particular case may appear. Conversely, the absence of alerts does not guarantee there are no aspects of the results needing attention. It is up to the individual to critically assess their own results and, if necessary, seek expert advice.

**Publication of your CIF in IUCr journals**

A basic structural check has been run on your CIF. These basic checks will be run on all CIFs submitted for publication in IUCr journals (*Acta Crystallographica*, *Journal of Applied Crystallography*, *Journal of Synchrotron Radiation*); however, if you intend to submit to *Acta Crystallographica Section C* or *E* or *IUCrData*, you should make sure that full publication checks are run on the final version of your CIF prior to submission.

**Publication of your CIF in other journals**

Please refer to the *Notes for Authors* of the relevant journal for any special instructions relating to CIF submission.

---

**PLATON version of 07/08/2019; check.def file version of 30/07/2019**

**Datablock aga_har-elmo** - ellipsoid plot

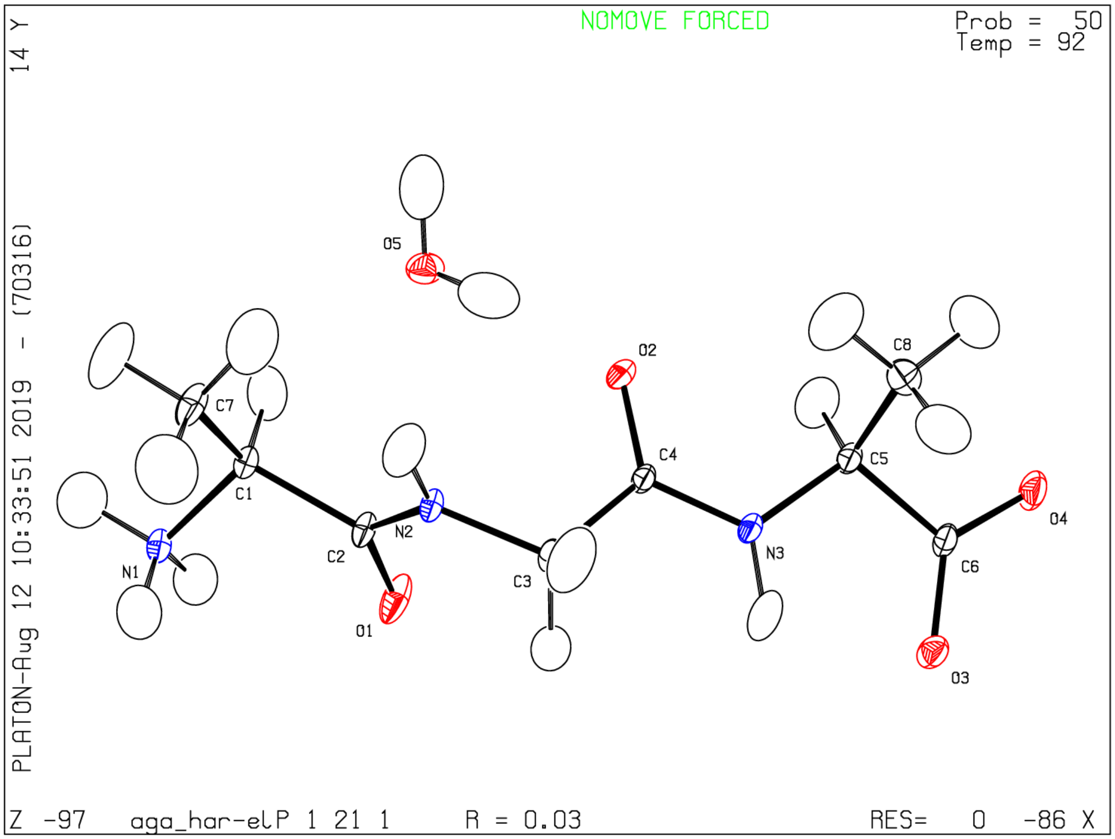

Supplement: Supplementary file 4 [file AGA_HAR-ELMO_checkcif.pdf › AGA_HAR-ELMO_checkcif.pdf]
